# Enhanced ORR Activity of Modified Recycled Graphite-Based Anode Materials

**DOI:** 10.1021/acsomega.5c13315

**Published:** 2026-03-11

**Authors:** Sukanya Sukanya, Kimia Hoseinzade, Frederik Bettels, Lin Zhang, René Wilhelm

**Affiliations:** † Institute of Organic Chemistry, 26534 Clausthal University of Technology, Leibnizstrasse 6, Clausthal-Zellerfeld 38678, Germany; ‡ Institute of Solid State Physics and Laboratory of Nano and Quantum Engineering, 26555 Leibniz University Hannover, Appelstrasse 2, Hannover 30167, Germany

## Abstract

Addressing the kinetic limitations of the oxygen reduction reaction (ORR) is essential for improving the efficiency of electrochemical energy-conversion devices such as fuel cells and metal–air batteries. Here, we demonstrate a circular-economy–oriented upcycling strategy for transforming end-of-life lithium-ion battery graphite anodes into metal-free ORR catalysts through oxidative activation and targeted molecular functionalization. Spent graphite anode material was activated by using H_2_SO_4_/HNO_3_ mixtures to increase defect density and surface reactivity, followed by surface functionalization with BPDI-OH-Cl, NDI-alendronic acid (NDI-ALEN), and NDI-aspartic acid (NDI-ASP). Acid activation significantly enhanced apparent ORR activity, yielding the highest half-wave potential (0.782 V for the 8 M acid-treated material), attributed to increased defect density and improved electrolyte accessibility. Subsequent molecular functionalization selectively modulated ORR behavior by introducing heteroatom-containing surface species, with NDI-ASP functionalization enhancing kinetic current density and charge-transfer characteristics relative to acid-treated graphite, although the half-wave potential remained slightly lower. XPS and SEM/EDX analyses confirm surface-confined incorporation of nitrogen- and phosphorus-containing molecular species following functionalization. These findings demonstrate that recycled graphite can serve as a chemically tunable, metal-free ORR catalyst platform, where defect generation governs apparent activity while molecular functionalization modulates kinetic behavior and effective electron-transfer characteristics, supporting circular-economy strategies for sustainable electrochemical energy conversion.

## Graphical abstract

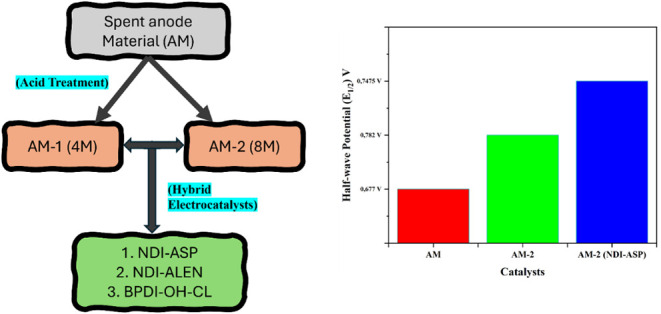

## 1 Introduction

The oxygen reduction reaction (ORR) is a key cathodic process in electrochemical energy-conversion devices such as proton exchange membrane fuel cells (PEMFCs) and metal–air batteries. Because ORR involves a complex four-electron transfer mechanism, it suffers from intrinsically slow kinetics, leading to high overpotentials and reduced system efficiency.^1^ As a result, the development of efficient, durable, and cost-effective ORR catalysts remains a central challenge in electrochemical energy research.^2,3^

Platinum-based catalysts are the current benchmark for the ORR due to their high activity and stability, particularly in acidic media.^4,5^ However, their large-scale deployment is limited by high cost, scarcity, and performance degradation caused by poisoning effects and fuel crossover.^6,7^ Carbon-based materials such as activated carbon and carbon black offer economic and environmental advantages but generally exhibit low intrinsic activity and limited durability under ORR conditions.^8^ Recent studies have shown that defect engineering and heteroatom incorporation in carbon materials can significantly enhance ORR activity and selectivity in alkaline media.^9^

In this context, increasing attention has been directed toward the upcycling of waste materials, especially spent graphite anodes from end-of-life lithium-ion batteries (LIBs). With the rapid growth of LIB consumption, sustainable strategies for valorizing spent anode material (AM)—particularly from NMC-type batteries—have become increasingly important. Rather than disposal, spent graphite can be converted into functional carbon materials through chemical activation and surface modification, supporting circular-economy principles while reducing environmental burden.^10^

Recent studies have demonstrated the feasibility of using recycled LIB graphite as a precursor for ORR electrocatalysts.^11^ Liivand et al. reported nitrogen-doped graphene derived from spent AM with promising ORR activity in alkaline media,^10^ while Ruan et al. developed heteroatom-doped recycled graphite catalysts for zinc–air batteries, achieving power densities of ∼100 mW cm^–2^.^12^ Despite these advances, recycled carbon catalysts often suffer from insufficient active site density and poorly optimized surface chemistry, limiting ORR performance.^13^

Graphene-based materials, such as graphene oxide (GO) and reduced graphene oxide (rGO) offer high surface area and tunable chemistry, making them attractive ORR platforms.^14–16^ However, pristine GO and rGO typically exhibit weak ORR activity without further activation or functionalization.^17^ Enhancing surface defects and introducing heteroatom-rich functional groups remain essential for improving ORR kinetics and selectivity, as widely demonstrated for nitrogen- and phosphorus-doped carbon catalysts.^18^

Herein, we report the activation of spent anode material (AM) using oxidative H_2_SO_4_/HNO_3_ mixtures (4 and 8 M), followed by surface functionalization with three organic molecules—BPDI-OH-Cl, NDI-alendronic acid (NDI-ALEN), and NDI-aspartic acid (NDI-ASP).^19^ These modifications aim to increase the defect density, introduce catalytically active sites, and tune ORR pathways. While strong mineral acids were employed in the present study, the sustainability motivation of this work is primarily rooted in circular-economy principles, namely the valorization of end-of-life battery graphite into metal-free electrocatalysts, rather than in strict adherence to green-chemistry process metrics at this stage.^6,20^ Accordingly, the current methodology is presented as a proof of concept for chemical upcycling, with future work directed toward reducing chemical intensity and improving process sustainability.

The electrocatalytic performance of the functionalized AM catalysts was systematically evaluated by using rotating disk electrode (RDE) measurements in alkaline media. Key ORR parameters—including onset potential (*E*_onset_), half-wave potential (*E*_1/2_), and diffusion-limited current density (*J*_L_)—were benchmarked against untreated AM and commercial carbon materials (GO, rGO, FG). The results demonstrate that organic functionalization effectively tunes the ORR selectivity toward a four-electron pathway, while apparent activity is governed by conductivity and accessible surface area. This study establishes a scalable, low-cost strategy for independently modulating ORR activity and pathway selectivity in metal-free catalysts derived from electronic waste, advancing sustainable materials development within a circular-economy framework.^21^ By decoupling defect-driven apparent activity from molecularly tuned ORR kinetics, this work establishes recycled graphite as a chemically tunable platform for metal-free ORR catalysis within a circular-economy framework.

## 2 Experimental Section

### 2.1 Materials and Reagents

Commercially available materials were used as received, including flake graphite (FG), graphene oxide (GO), and reduced graphene oxide (rGO). Spent anode material (AM), originating from NMC 111-type lithium-ion batteries, was supplied by a collaborative partner at TU Braunschweig. The AM was manually extracted from disassembled battery cells and subjected to an initial wet-chemical purification process to remove extraneous battery components prior to acid activation. BPDI-OH-Cl, bromohydrin (BrOH), aspartic acid, alendronic acid, and Amberlyst IRA-900 resin were used as received without further purification.

### 2.2 Acid Treatment of the Spent Anode Material (AM)

Acid treatment of the spent anode material (AM) was carried out to modify its surface chemistry and remove residual impurities. Two different acid treatment conditions were employed to assess the effect of oxidative strength on the material’s properties, following similar protocols reported for graphite oxidation and purification.^19^ In each case, 16.0 g of AM was dispersed in a 150 mL mixture of concentrated sulfuric acid (H_2_SO_4_, 98%) and nitric acid (HNO_3_, 65%) at defined molarities of 4 and 8 M, respectively. The acid mixtures were prepared in a 3:1 volumetric ratio (H_2_SO_4_:HNO_3_) and added to a 500 mL three-neck round-bottom flask equipped with a reflux condenser, magnetic stirrer, and temperature controller. The suspension was stirred vigorously and subjected to thermal treatment in an oil bath maintained at 90 °C for 24 h under reflux conditions. The oxidation process was monitored visually by darkening of the suspension and release of nitrogen oxides. Upon completion, the reaction mixtures were cooled to room temperature and filtered by using vacuum filtration. The recovered solids were repeatedly washed with DI water until the filtrate reached near-neutral pH, followed by additional rinsing with a dilute NaOH solution to ensure complete neutralization. The washed products were dried in an oven at 80–90 °C overnight. The treated samples were labeled according to the acid concentration used: AM-1 (4 M) and AM-2 (8 M). Both samples were stored in airtight glass containers to prevent moisture uptake and preserve their chemical integrity for subsequent analytical measurements. The material was subsequently characterized to evaluate its morphology, elemental composition, and electrochemical behavior, following characterization protocols established in similar graphite recovery studies.^19^ This acid activation step was employed to establish a reproducible, defect-rich carbon surface for subsequent comparative functionalization studies rather than as an optimized low-impact processing route of spent anode material (AM) recovered from disassembled NCM 111-type lithium-ion batteries. The AM was subjected to acid treatment using H_2_SO_4_/HNO_3_ (3:1) under reflux conditions to yield two treated samples (AM-1 and AM-2). The resulting powders were formulated into catalyst inks for electrochemical testing by using a three-electrode configuration. Post-treatment surface modification with organic molecules—NDI-aspartic acid (NDI-ASP), NDI-alendronic acid (NDI-ALEN), and BPDI-OH-Cl—was explored to investigate molecular grafting effects on ORR performance. The experimental procedure for the processing and evaluation is illustrated in Figure 1.

**Figure 1:**
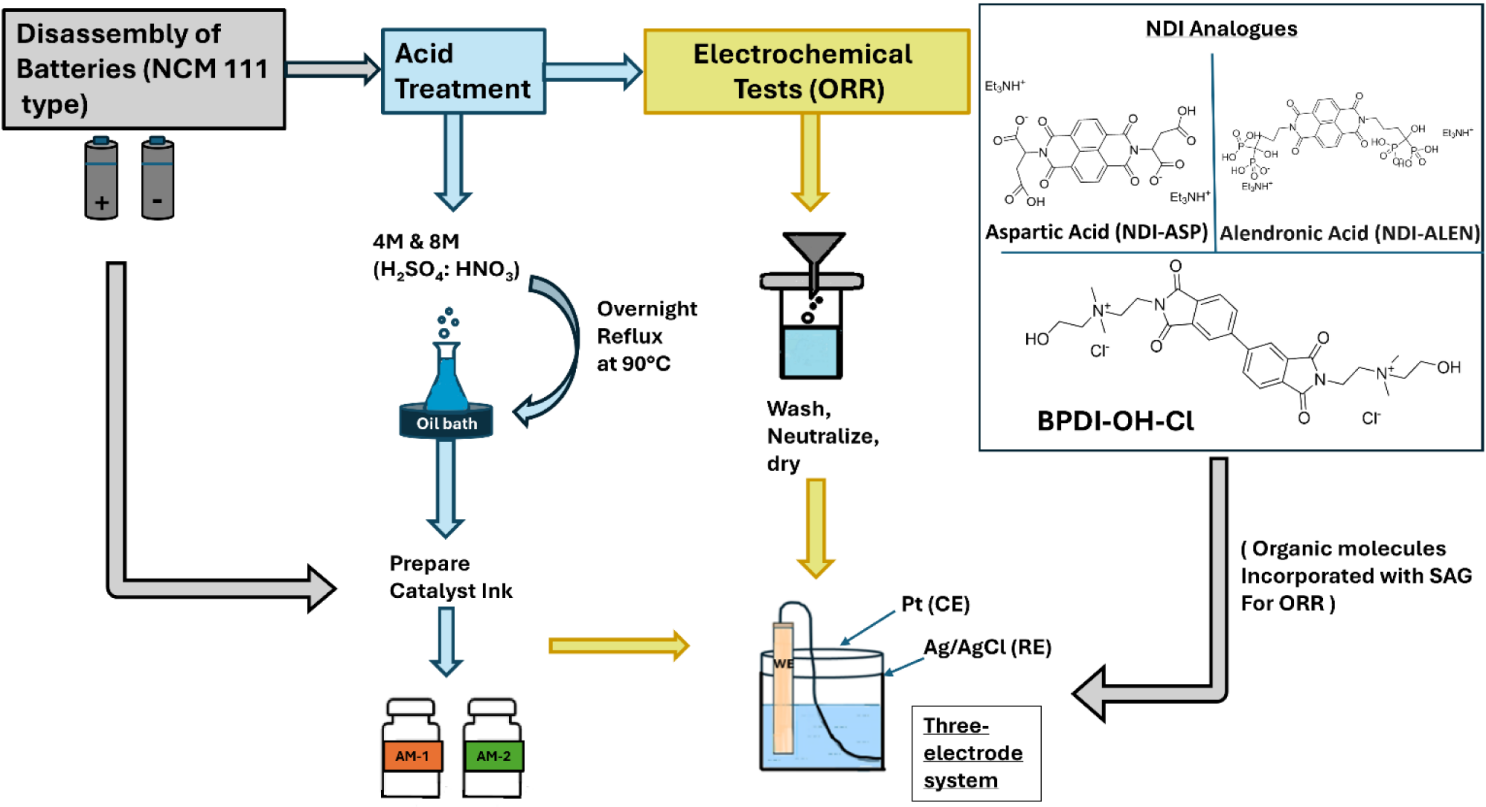
Schematic illustration of the experimental procedure for processing and evaluation.

#### 2.2.1 Functionalization Process

##### 2.2.1.1 2,2′-Bis­(2-(dimethylamino)­ethyl)-[5,5′-biisoindoline]-1,1′,3,3′-tetraone (BPDI)


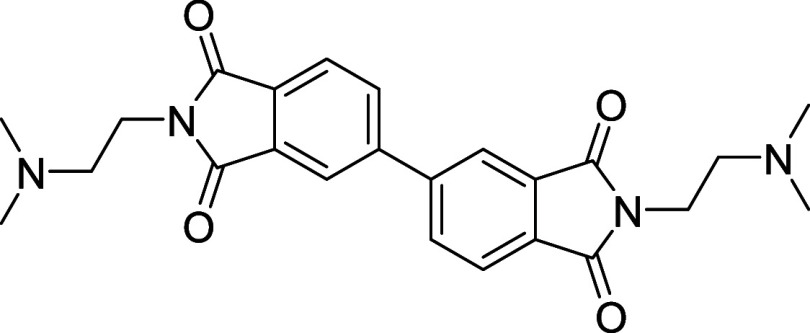


To a stirred solution of 3,3′,4,4′-biphenyltetracarboxylic dianhydride (0.02 mol, 5.88 g) in toluene (150 mL) at 90 °C was added dropwise *N*,*N*-dimethylethylenediamine (0.05 mol, 4.41 g) in toluene (50 mL) over 10 min. The mixture was stirred for 60 min at 90 °C and then heated to 130 °C under a Dean–Stark trap overnight. After the mixture was cooled to room temperature, the white solid was collected by filtration, washed with water, and dried under high vacuum to afford the product (0.00984 mol, 4.28 g, 49.2% yield). ^**1**^ **H NMR (400 MHz, CDCl_3_)**: δ 8.01 (s, 2H), 7.88 (t, 4H), 3.76 (t, 4H), 2.54 (t, 4H), 2.21 (s, 12H). ^13^C NMR (100 MHz, CDCl_3_): δ 36.23, 45.54, 57.08, 122.06, 124.04, 131.96, 132.78, 133.40, 145.09, 167.84. HRMS: *m*/*z* = 435.20 [M + H]^+^.

##### 2.2.1.2 2,2′-(1,1′,3,3′-Tetraoxo-[5,5′-biisoindoline]-2,2′-diyl)­bis­(*N*-(2-hydroxyethyl)-*N*,*N*-dimethylethan-1-aminium) Bromide (BPDI-OH-Br)


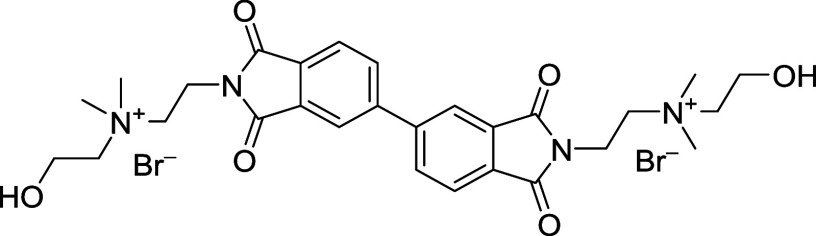


A mixture of BPDI (0.005 mol, 2.172 g), bromohydrin (0.01 mol, 1.24 g), and dry acetonitrile (15 mL) was heated to 90 °C under nitrogen and stirred overnight. The solvent was removed under reduced pressure, and the residue was dried under high vacuum to afford the product (0.00471 mol, 3.21 g, 94.1% yield). ^**1**^ **H NMR (400 MHz, D**_**2**_ **O + TMSP-*d***_**4**_ **)**: δ 7.90 (m, 6H), 4.26 (t, 4H), 4.19 (t, 4H), 3.84 (t, 4H), 3.74 (m, 4H), 3.40 (s, 12H), 3.08 (m, 2H). ^13^C NMR (100 MHz, D_2_O + TMSP-*d*_4_): δ 34.56, 54.92, 58.31, 63.95, 68.66, 125.17, 127.09, 133.57, 134.82, 136.61, 147.32, 171.29, 171.49. HRMS: *m*/*z* = 262.13 [M – 2Br/2]^+^.

##### 2.2.1.3 2,2′-(1,1′,3,3′-Tetraoxo-[5,5′-biisoindoline]-2,2′-diyl)­bis­(*N*-(2-hydroxyethyl)-*N*,*N*-dimethylethan-1-aminium) Chloride (BPDI-OH-Cl)


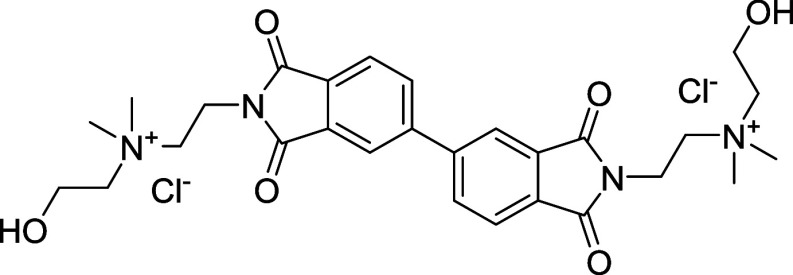


BPDI-OH-Br (1 g) was dissolved in deionized water (10 mL) and then passed through an ion-exchange column (Amberlyst IRA-900 resin) to replace Br^–^ with Cl^–^. The solvent was removed under reduced pressure, and the product was dried under high vacuum (0.0013 mol, 0.79 g, 90.4% yield). ^**1**^ **H NMR (400 MHz, D**_**2**_ **O + TMSP-*d***_**4**_ **)**: δ 7.90 (m, 6H), 4.28 (t, 4H), 4.21 (t, 4H), 3.86 (t, 4H), 3.76 (t, 4H), 3.42 (s, 12H), 3.09 (m, 2H). ^13^C NMR (100 MHz, D_2_O+TMSP-*d*_4_): δ 34.54, 45.99, 46.14, 54.91, 58.30, 63.91, 68.66, 125.14, 127.06, 133.54, 134.79, 136.58, 147.27, 171.25, 171.46. HRMS: *m*/*z* = 262.13 [M – 2Cl/2]^+^.

##### 2.2.1.4 2,2′-(1,3,6,8-Tetraoxo-1,3,6,8-tetrahydrobenzo­[*lmn*]­[3,8]­phenanthroline-2,7-diyl)­bis­(3-carboxypropanoate) Triethylammonium (NDI-ASP)


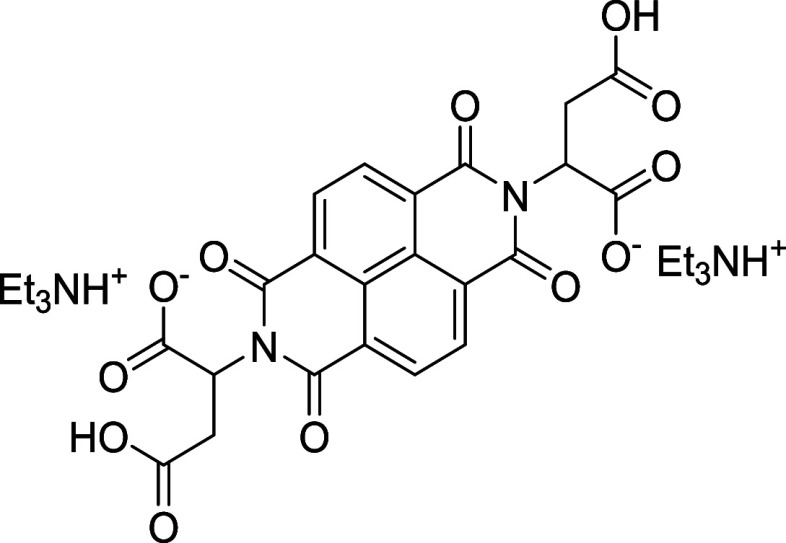


To a solution of 1,4,5,8-naphthalenetetracarboxylic dianhydride (0.037 mol, 10 g) in DMF (100 mL) was added NDI-aspartic acid (0.077 mol, 10.34 g) and triethylamine (0.40 mol, 56.2 mL). The mixture was heated to 115 °C for 16 h under air. A green solid precipitated over time. The mixture was filtered, the solid washed with diethyl ether, and dried under high vacuum (0.022 mol, 16.02 g, 61.3% yield). ^**1**^ **H NMR (400 MHz, D**_**2**_ **O + TMSP-*d***_**4**_ **)**: δ 8.71 (s, 4H), 5.93 (q, 2H), 3.45 (q, 2H), 3.24 (q, 12H), 2.90 (q, 2H), 1.32 (t, 18H)). ^13^C NMR (100 MHz, D_2_O + TMSP-*d*_4_): δ 8.20, 35.42, 46.61, 52.32, 126.21, 131.25, 163.71, 174.50, 176.06. HRMS (negative mode): *m*/*z* = 497.04 [M – H]^+^.

##### 2.2.1.5 ((1,3,6,8-Tetraoxo-1,3,6,8-tetrahydrobenzo­[*lmn*]­[3,8]­phenanthroline-2,7-diyl)­bis­(1-hydroxy-1-phosphonobutane-4,1-diyl))­bis­(hydrogen phosphonate) Triethylammonium (NDI-ALEN)


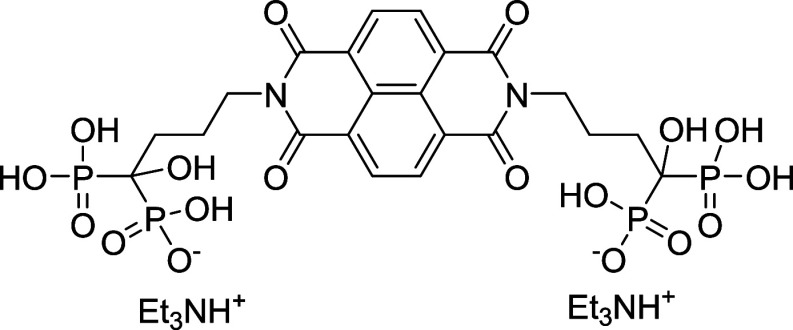


To a solution of 1,4,5,8-naphthalenetetracarboxylic dianhydride (0.014 mol, 4.00 g) in DMF (100 mL) was added NDI-alendronic acid (0.029 mol, 7.37 g) and triethylamine (0.20 mol, 28 mL). The mixture was heated to 140 °C for 16 h under air. A yellow solid precipitated over time. The mixture was filtered, washed with diethyl ether, dissolved in water, and filtered to remove insoluble residues. The aqueous phase was concentrated under reduced pressure and dried under high vacuum to give the product (0.0058 mol, 11.09 g, 79.7% yield). ^**1**^ **H NMR (400 MHz, D**_**2**_ **O + TMSP-*d***_**4**_ **)**: δ 8.39 (s, 4H), 4.089 (t, 4H), 3.18 (q, 16H), 2.02 (m, 8H), 1.2 (t, 24H)). ^13^C NMR (100 MHz, D_2_O + TMSP-*d*_4_): δ 11.22, 25.27, 33.83, 49.60, 76.48, 128.30, 128.65, 133.88, 166.60. HRMS (negative mode): *m*/*z* = 729.0058 [M – H]^+^.

### 2.3 Post-treatment Surface Functionalization of Spent Anode Material (AM)

To further enhance the catalytic activity, the acid-treated samples (AM-1 and AM-2) were modified with organic molecules. The purified spent anode material (AM) powder was dispersed in deionized water (5 mg mL^–1^) and sonicated for 30 min. Functional molecules—NDI-aspartic acid (NDI-ASP), NDI-alendronic acid (NDI-ALEN), or BPDI-OH-Cl—were added in nominal stoichiometric ratios based on relative surface oxygen functionalities inferred from XPS analysis rather than on absolute quantification of surface −OH/–COOH densities. The suspensions were stirred at room temperature for 12 h to promote surface adsorption. The resulting hybrid materials were collected and washed thoroughly with deionized water to remove unbound molecules, followed by drying at 90 °C overnight. These functionalization conditions promote molecular grafting onto the acid-activated carbon surface primarily through noncovalent interactions (e.g., π–π interactions, hydrogen bonding, and electrostatic interactions) with possible defect-mediated attachment. Direct quantification of molecular loading (wt % or surface coverage) was not performed; all functionalization reactions were conducted under identical conditions to enable meaningful comparative assessment of ORR performance.

### 2.4 Electrochemical Measurements

The oxygen reduction reaction (ORR) performance of the prepared catalysts was evaluated by using a conventional three-electrode configuration connected to an Autolab PGSTAT204 potentiostat/galvanostat (Metrohm). A platinum wire served as the counter electrode, and an Ag/AgCl (saturated KCl) electrode was used as the reference electrode. All measured potentials were converted to the reversible hydrogen electrode (RHE) scale using

$$E_{\mathrm{RHE}}=E_{\mathrm{Ag}/\mathrm{AgCl}}+0.059\times \mathrm{pH}+0.197\;V,(\mathrm{in}\;0.1\;M\;\mathrm{KOH})$$

for measurements in 0.1 M KOH.

To prepare the catalyst ink, 5 mg of catalyst powder was dispersed in a mixture consisting of 450 μL of deionized water, 50 μL of ethanol, and 125 μL of a 0.05 wt % Nafion solution. The suspension was sonicated for 2 h to obtain a homogeneous and stable dispersion. A 20 μL aliquot of the resulting ink was drop-cast onto a polished glassy carbon (GC) disk electrode (5 mm diameter, geometric area = 0.196 cm^2^) and dried under ambient conditions. The deposited catalyst mass was 0.16 mg, corresponding to a catalyst loading of 0.82 mg cm^–2^ on the glassy carbon electrode (geometric area = 0.196 cm^2^). This loading was used consistently for all CV, LSV, and RDE measurements.

Cyclic voltammetry (CV) was conducted in the potential range of 1.2 to −1.2 V vs RHE at a scan rate of 50 mV s^– 1^ to assess background capacitive behavior and general electrochemical characteristics of the catalyst layers. ORR-related parameters, including onset potential (*E*_onset_) and half-wave potential (*E*_1/2_), were determined exclusively from the positive potential region (≥∼0.2 V vs RHE), where oxygen reduction occurs. The negative potential region, associated with hydrogen evolution, was not considered in the ORR analysis. Diffusion-limited current densities and mass-transport behavior were obtained from LSV/RDE measurements conducted within a conventional ORR potential window.

Rotating disk electrode (RDE) experiments were performed by using linear sweep voltammetry (LSV) over the same potential range at a scan rate of 10 mV s^–1^. Electrode rotation speeds were varied between 400 and 2500 rpm to evaluate mass-transport effects and ORR kinetics. The diffusion-limited current density (*J*_L_) and kinetic parameters were obtained exclusively from the LSV/RDE data sets. The electrochemical behavior of the chemically treated AM-1 and AM-2 materials was examined before and after organic surface functionalization with NDI-aspartic acid (NDI-ASP), NDI-alendronic acid (NDI-ALEN), and BPDI-OH-Cl. Their ORR performance was benchmarked against reference carbon materials, including spent anode material (AM), graphene oxide (GO), flake graphite (FG), and reduced graphene oxide (rGO).

### 2.5 Catalyst Characterizations

The structural, compositional, and defect characteristics of the recycled graphite-based materials were evaluated using Raman spectroscopy, X-ray diffraction (XRD), and X-ray fluorescence (XRF) to assess the effects of acid leaching on the spent anode material.^19^ Surface-sensitive elemental analysis was conducted using X-ray photoelectron spectroscopy (XPS) on a PHI VersaProbe III Scanning ESCA system equipped with an RH-anode X-ray source operated at 40 kV. Scanning electron microscopy (SEM) was performed by using an in-lens secondary electron (SE) detector at an accelerating voltage of 4.0 kV to characterize surface morphology at high resolution. Energy-dispersive X-ray spectroscopy (EDX) was conducted by using a Bruker Esprit 1.82 system with an accelerating voltage of 20 kV to obtain elemental distribution information.

## 3 Results and Discussion

Disassembly of NCM 111-type lithium-ion batteries, followed by acid treatment using sulfuric and nitric acid mixtures of two different concentrations (4 and 8 M H_2_SO_4_:HNO_3_), enabled effective removal of transition-metal-containing species from the spent anode material.^22^ As shown in Figure 2, SEM micrographs of the acid-treated samples exhibit clear differences in surface morphology as a function of the acid concentration. The AM-1 sample (4 M acid treatment) retained a relatively dense and layered particle structure, consistent with partial leaching of inorganic species. In contrast, the AM-2 sample (8 M acid treatment) displayed a markedly more porous and fragmented microstructure, suggesting more aggressive dissolution of Ni-, Co-, Mn-, and Li-containing phases and partial disruption of the graphite matrix.^22,23^ These morphological observations were supported by EDX analysis, which confirmed the depletion of transition metals from the bulk, with the 8 M treatment showing lower residual metal content.^23^ These observations were corroborated by EDX analysis, which demonstrated a lower residual metal content for AM-2 material. The enhanced porosity and increased surface roughness imparted by the higher acid concentration are expected to expose additional active sites and improve ion accessibility—both of which are beneficial for catalytic processes.

**Figure 2:**
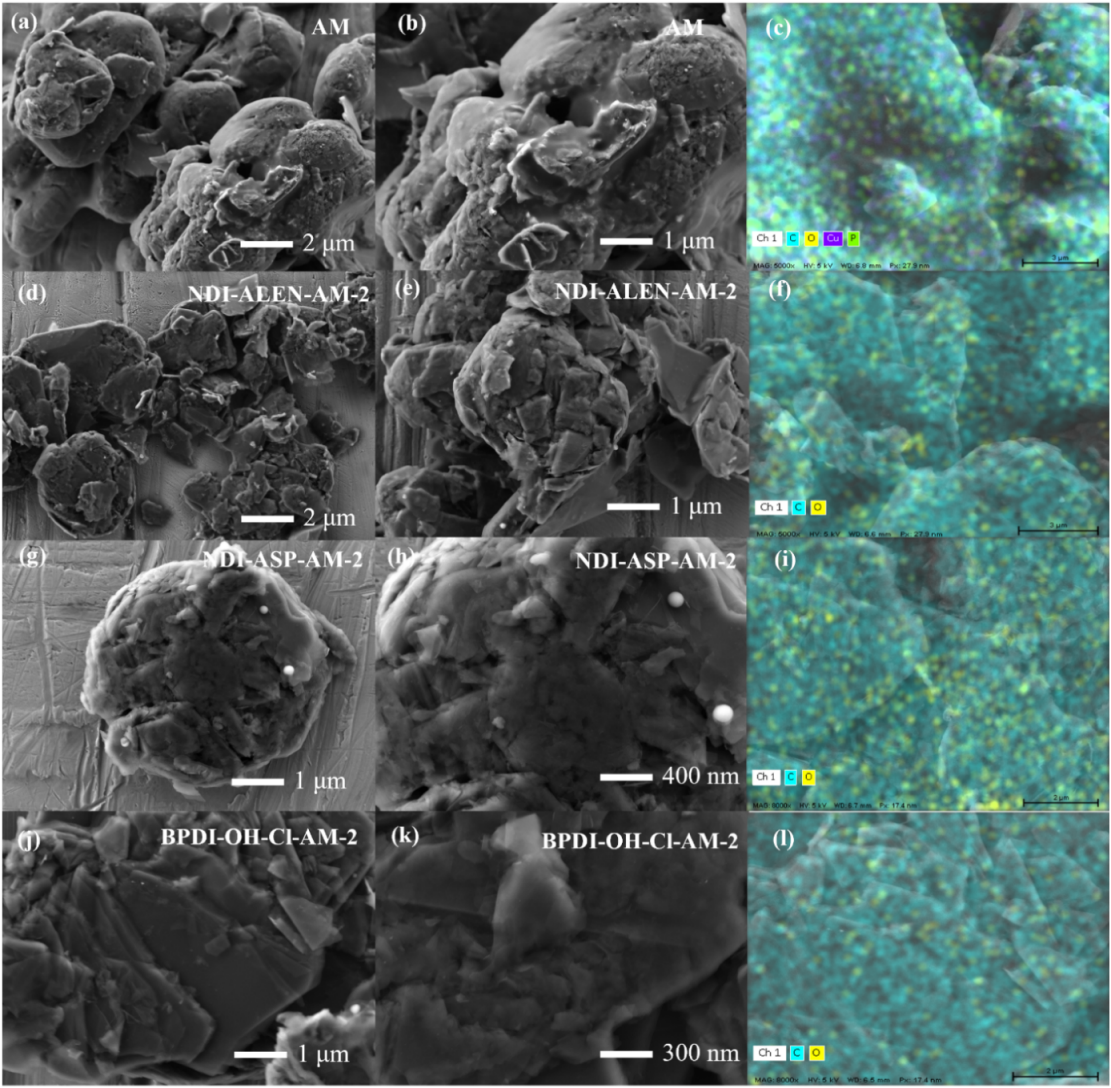
SEM images and corresponding EDX elemental maps of untreated and functionalized spent graphite samples. (a, b) SEM micrographs of untreated spent anode material (AM) at different magnifications, showing relatively smooth graphite particles. (c) EDX map of AM confirming dominant carbon with minor oxygen content. (d, e) SEM images of NDI-ALEN–AM-2 displaying increased surface roughening and fragmentation after acid activation and functionalization. (f) Corresponding EDX map indicating incorporation of heteroatom-containing species. (g, h) SEM images of NDI-ASP–AM-2 revealing defect-rich, roughened morphology. (i) EDX map showing homogeneous elemental distribution. (j, k) SEM images of BPDI-OH-Cl–AM-2 illustrating stacked platelet-like features following modification. (l) Corresponding EDX map confirming surface elemental distribution. Bulk compositions are summarized in Table S1 (Supporting Information).

To further enhance the ORR activity, the recovered materials were functionalized with three organic molecules: NDI-aspartic acid (NDI-ASP), NDI-alendronic acid (NDI-ALEN), and BPDI-OH-Cl. These compounds were selected based on their distinct electronic and structural features.

- NDI-ASP contains amine and carboxylate groups, offering nitrogen incorporation and providing potential sites for hydrogen bonding or metal chelation.^24^
- NDI-ALEN introduces phosphonate groups, which interact strongly with oxidized surfaces and can form stable surface complexes.^25^
- BPDI-OH-Cl contains electron-donating groups and an extended π-conjugated system that promotes charge delocalization and enhances redox behavior.

The NDI and BPDI macro-organic molecules were selected due to their electron-deficient π-systems, which enable modulation of the surface chemistry of recycled graphite. When grafted onto oxygen-rich defect sites generated during acid activation, these molecules introduce nitrogen- and oxygen-containing functionalities and locally polarized surface environments that facilitate oxygen adsorption and improve electrolyte interaction. The resulting increase in surface heteroatom content and wettability provides a mechanistic basis for the observed enhancement of the ORR benchmarking parameters. The organic molecules are grafted onto the oxidized graphite surface through a combination of noncovalent interactions (π–π stacking, hydrogen bonding, and electrostatic interactions) and possible defect-mediated attachment. While surface-associated bonding at defect sites is chemically plausible, the present XPS data do not provide definitive evidence for new C–N or C–P bond formation. The surface morphology and elemental distribution of the untreated and functionalized materials were investigated by SEM and EDX. As illustrated in Figure 2, the untreated spent anode material consists of densely packed agglomerates with relatively smooth graphitic regions, a morphology typical of aged lithium-ion battery anodes. After acid activation and subsequent functionalization, discernible yet moderate changes in the surface texture are observed. The NDI-ALEN-functionalized AM-2 sample shows more disrupted and partially fractured surface regions, consistent with oxidative modification of the outer graphite layers.^25^ The NDI-ASP-functionalized AM-2 material exhibits a roughened surface with localized features, suggesting an increased density of defect sites.^24^ In contrast, BPDI-OH-Cl (AM-2) largely retains planar graphitic characteristics but displays thinner, plate-like fragments, indicative of more uniform surface etching. Although the overall particle morphology remains largely preserved—as expected for highly graphitized carbon—the observed variations are consistent with defect formation that facilitates subsequent surface functionalization.

EDX elemental mapping reveals a homogeneous spatial distribution of carbon and oxygen across the functionalized samples, supporting uniform surface oxidation. Because EDX is inherently limited in sensitivity to light elements present at low concentrations on the surface, quantitative elemental analysis was not pursued. Instead, surface heteroatom incorporation was assessed primarily by XPS, which shows increases in the N 1s, O 1s, and P 2p signals following functionalization.^24,25^

Additional information about surface composition was obtained from X-ray photoelectron spectroscopy (XPS), as presented in Figure 3. The high-resolution C 1s spectrum of the untreated spent anode material (AM) sample contains contributions associated with graphitic carbon and oxidized carbon species, consistent with a partially disordered graphite surface containing oxygenated functionalities.^26,27^ After functionalization, all modified samples [NDI-ALEN (AM-2), NDI-ASP (AM-2), and BPDI-OH-Cl (AM-2)] display increased contributions from oxygen- and nitrogen-containing surface species, confirming effective surface modification.^28,29^ The appearance of N and P signals after functionalization confirms successful surface modification, although XPS atomic percentages were not converted to absolute molecular loading values. The O 1s spectra exhibit features corresponding to C–O, C=O, and −OH groups, with relative contributions dependent on the specific ligand employed. Notably, the NDI-ASP (AM-2) and BPDI-OH-Cl (AM-2) samples show more pronounced O–C=O and −OH components, consistent with the development of more hydrophilic and chemically accessible surfaces, as shown in Table 1.^28^

**Table 1:** X-ray Photoelectron Spectroscopy (XPS) Atomic Concentrations (At%) for Samples Spent Anode Material (AM), NDI-ASP (AM-2), NDI-ALEN (AM-2), and BPDI-OH (AM-2), Calculated Using Instrument-Specific Relative Sensitivity Factors

Parameter/Sample	C 1s	N 1s	O 1s	P 2p
RSF	0.314	0.499	0.733	0.525
Corrected RSF	67.330	106.033	153.608	130.875
AM (at%)	70.00	0.39	28.41	1.21
AS-1 (at%)	86.36	1.92	11.72	0.00
AA-1 (at%)	86.68	1.64	11.64	0.04
P-1 (at%)	86.64	1.07	12.29	0.00

**Figure 3:**
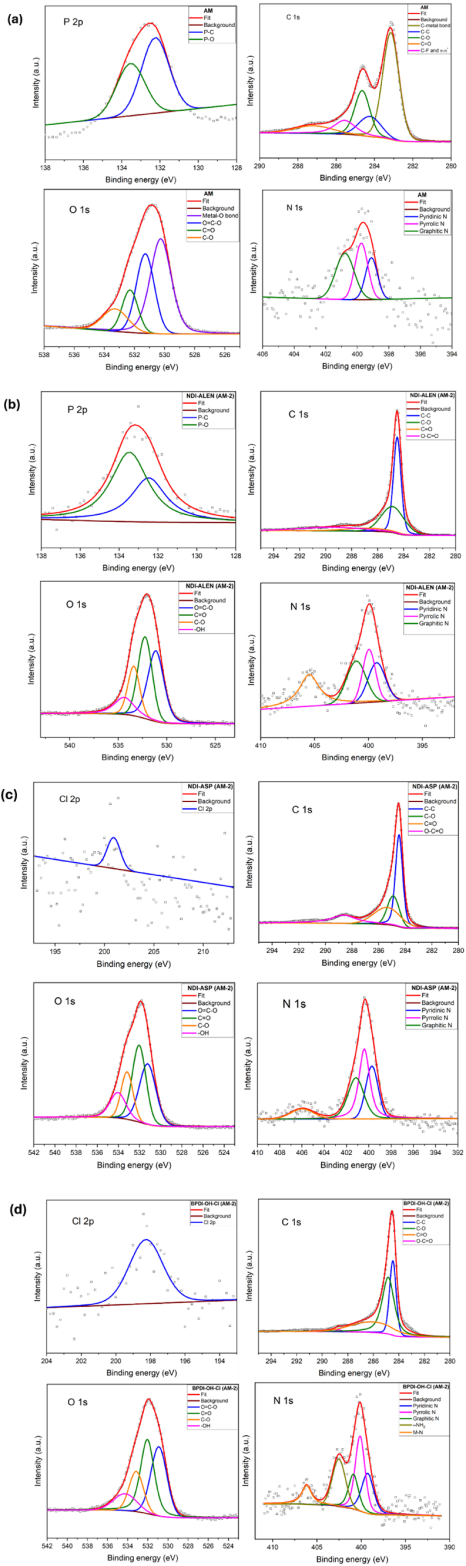
High-resolution XPS spectra of graphite samples before and after acid treatment: (a) Spent anode material (AM); (b) NDI-ALEN (AM-2); (c) NDI-ASP (AM-2); (d) BPDI-OH-Cl (AM-2). Spectra include P 2p, C 1s, O 1s, N 1s, and Cl 2p regions, fitted with their corresponding qualitative fitting. Deconvolution indicates the presence of phosphate species (P–O and P=O), various carbon functionalities (C–C, C–O, C=O, and O–C=O), oxygen species (C=O, O–C=O, C–O, and OH), and nitrogen configurations (pyridinic, pyrrolic, and graphitic N), as well as trace chloride incorporation. These profiles reveal the chemical modifications and bonding environments introduced by each treatment method.

Nitrogen-containing surface species detected in the N 1s region are consistent with nitrogen functionalities commonly reported for modified carbon materials and are known to promote oxygen adsorption and electron transfer during ORR.^30^ Pyridinic- and graphitic-type nitrogen environments have been widely associated with enhanced oxygen adsorption and improved electron-transfer characteristics.^6,8^ Phosphorus-containing species detected in the NDI-ALEN (AM-2) sample are consistent with the incorporation of phosphonate moieties derived from alendronic acid.^31^ Low-intensity Cl 2p signals observed in the NDI-ASP (AM-2) and BPDI-OH-Cl (AM-2) samples originate from residual chlorine-containing precursors. Tables 1 and 2, together with bulk EDX data provided in Table S1, show that acid-activated samples, followed by molecular functionalization, alter the carbon surface chemistry and introduce catalytically relevant heteroatoms (N, P, and O), thereby collectively enhancing the ORR performance. As expected, nitrogen- and phosphorus-containing species are detectable by surface-sensitive XPS but remain below the detection limit of bulk-sensitive EDX.

**Table 2:** Comparison of Surface (XPS) and Bulk (EDX) Elemental Compositions of Recycled Graphite Samples

Sample	Technique	C (at%)	N (at%)	O (at%)	P (at%)	Cu (at%)
AM	XPS	70.00	0.39	28.41	1.21	—
	EDX	96.07	—	3.33	—	0.60
AS-1	XPS	86.36	1.92	11.72	0.00	—
	EDX	98.28	—	1.72	—	—
AA-1	XPS	86.68	1.64	11.64	0.04	—
	EDX	98.01	—	1.99	—	—
P-1	XPS	86.64	1.07	12.29	0.00	—
	EDX	99.30	—	0.70	—	—

Although high-resolution XPS confirms the presence of N- and P-containing surface species after functionalization, the spectra do not provide unambiguous evidence for newly formed surface-associated C–N or C–P bonds; therefore, covalent surface bonding cannot be conclusively assigned based on XPS alone. Although Brunauer–Emmett–Teller (BET) surface area analysis and Fourier-transform infrared (FTIR) spectroscopy were not performed in this study, their absence does not preclude comparative interpretation of the observed material transformations under identical electrochemical testing conditions. BET surface area measurements and electrochemically active surface area estimates were unavailable; therefore, the ORR current densities are reported per geometric electrode area and are not surface-area normalized. Accordingly, the observed electrochemical trends reflect the combined effects of surface chemistry, defect density, electrical conductivity, and accessible porosity rather than the intrinsic site-specific activity. FTIR spectroscopy is also of limited utility for highly graphitic materials due to weak IR-active vibrations and strong background absorption. Structural and chemical evolution of the materials is nevertheless well supported by complementary characterization techniques, including X-ray diffraction (XRD), Raman spectroscopy, scanning electron microscopy with energy-dispersive X-ray spectroscopy (SEM/EDX), and X-ray photoelectron spectroscopy (XPS). In addition, bulk elemental analysis using X-ray fluorescence (XRF) and total reflection X-ray fluorescence (TXRF) was employed to assess residual metal impurities, following methodologies established in our related work on similarly processed spent anode materials.^32^ Acid treatment increases defect density and oxygen-containing surface functionalities, as evidenced by increased Raman D/G ratios and XPS elemental trends, in agreement with previous reports on oxidatively treated carbon materials and recycled graphite.^33^ Taken together, these complementary characterization techniques provide a coherent basis for interpreting the enhanced ORR performance of the functionalized catalysts.^33^ BET analyses will be incorporated in future work to further quantify the porosity and surface functional groups when appropriate instrumentation becomes available. Moreover, the combination of XPS elemental trends and electrochemical performance data is sufficient to establish a correlation between surface functionalization and improved catalytic behavior in the present study. Consistent with its bulk sensitivity, EDX does not detect surface-confined heteroatoms (Table S1), confirming that nitrogen- and phosphorus-containing species introduced during functionalization are confined to the surface of the recycled graphite.

### 3.1 Electrochemical Activity (ORR)

The ORR performance of commercial carbon materials and recycled spent anode material was first evaluated to establish a baseline for comparison. Cyclic voltammetry (CV) and linear sweep voltammetry (LSV) measurements were conducted in O_2_- and N_2_-saturated 0.1 M KOH. All samples exhibited negligible cathodic current under N_2_, while pronounced reduction currents were observed under O_2_, confirming the ORR activity (Figures 4–8). Koutecký–Levich analysis and electron-transfer numbers are discussed only for catalysts exhibiting linear K–L behavior; for samples showing mixed kinetic–diffusion control, quantitative *n* values were not extracted to avoid overinterpretation. Among the commercial benchmarks, graphene oxide (GO) showed the most positive ORR initiation, with an onset potential (*E*_onset_) of 0.873 V and a half-wave potential (*E*_1/2_) of 0.754 V versus RHE, consistent with its high surface oxygen content and favorable wettability. Reduced graphene oxide (rGO) displayed moderate ORR activity, reflecting improved electronic conductivity relative to GO but fewer oxygen-containing surface functionalities.^34^ In contrast, untreated spent anode material (AM) exhibited significantly lower activity (*E*_onset_ = 0.751 V; *E*_1/2_ = 0.677 V; *J*_L_ = 2.14 mA cm^– 2^), indicative of kinetically constrained ORR behavior on the recycled graphite surface. All electrochemical measurements were conducted by using identical catalyst loadings, electrode geometry, rotation rates, and electrolyte composition to ensure meaningful comparison across samples. Because BET surface area measurements were not available, the ORR current densities are not normalized to the surface area and therefore reflect combined effects of surface chemistry, defect density, conductivity, and accessible porosity rather than intrinsic site-specific activity.

**Figure 4:**
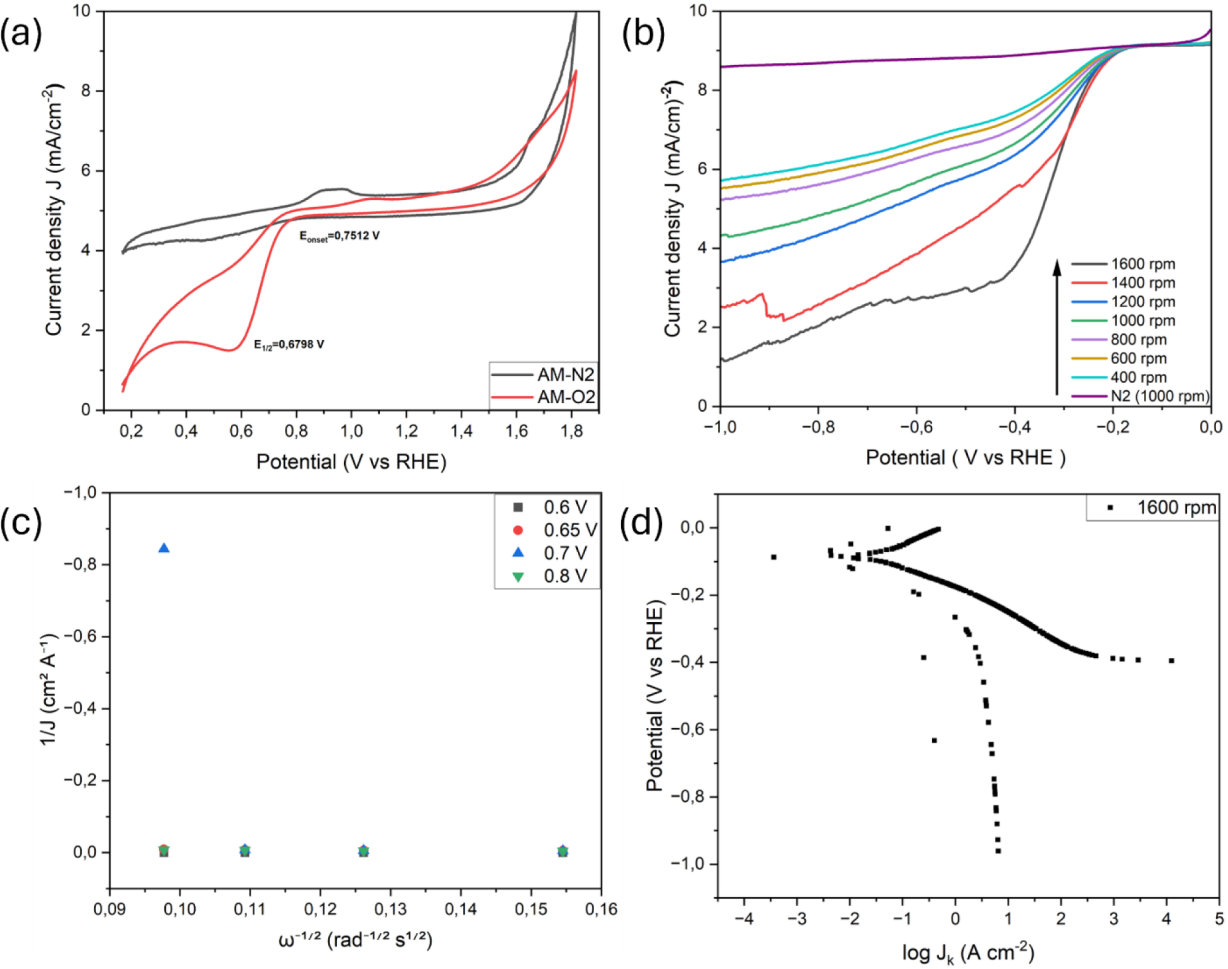
ORR response of untreated spent anode material (AM), (a) Cyclic voltammograms of untreated AM in O_2_- and N_2_-saturated 0.1 M KOH, (b) RDE polarization curves collected at varying rotation speeds, and (c) Koutecký–Levich plots showing deviation from linearity, reflecting kinetically limited ORR behavior. *Note: “All electrochemical measurements were performed using identical catalyst loadings (0.82 mg cm^– 2^), electrode geometry, and electrolyte composition.”*

**Figure 5:**
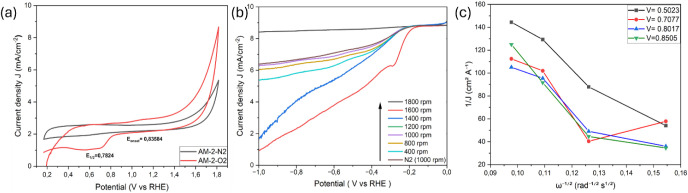
ORR activity of 8 M acid-treated spent anode material (AM-2): (a) CVs of AM-2 measured in O_2_- and N_2_-saturated 0.1 M KOH, (b) RDE polarization curves at different rotation rates (400–1800 rpm), and (c) Koutecký–Levich plots derived from the RDE data, exhibiting near-linear behavior consistent with diffusion-dominated ORR.

**6:**
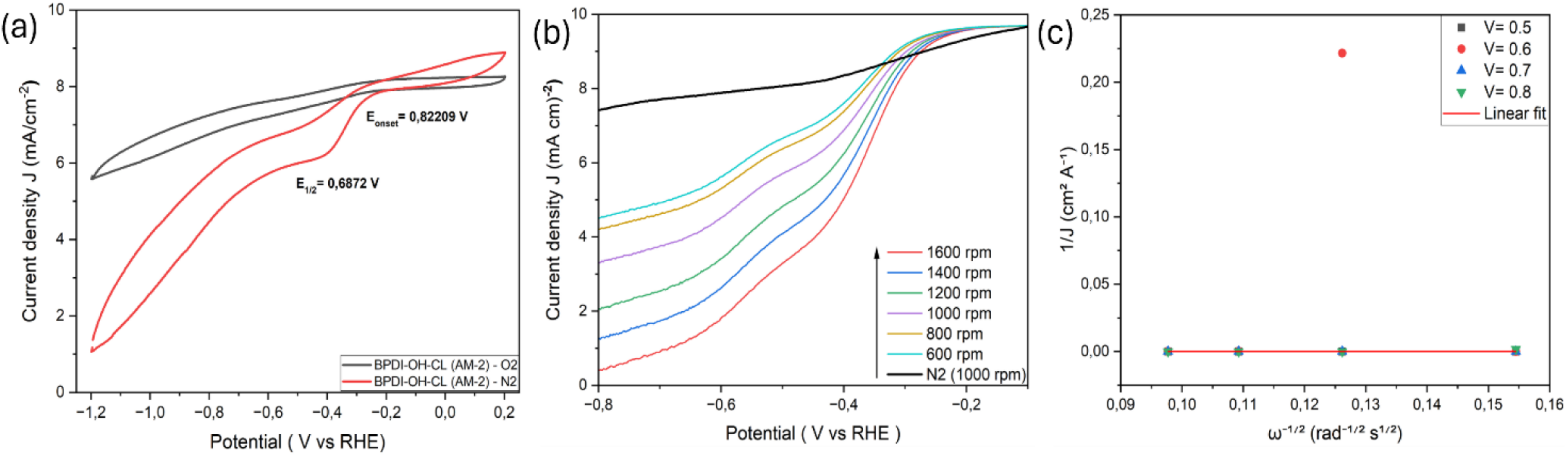
BPDI-OH-Cl–functionalized AM-2 ORR performance: (a) Cyclic voltammograms of BPDI-OH-Cl (AM-2) in O_2_- and N_2_-saturated 0.1 M KOH, (b) RDE polarization curves recorded at rotation rates from 400 to 1600 rpm, and (c) corresponding Koutecký–Levich plots at selected potentials, showing nonideal linearity indicative of mixed kinetic–diffusion control.

**Figure 7:**
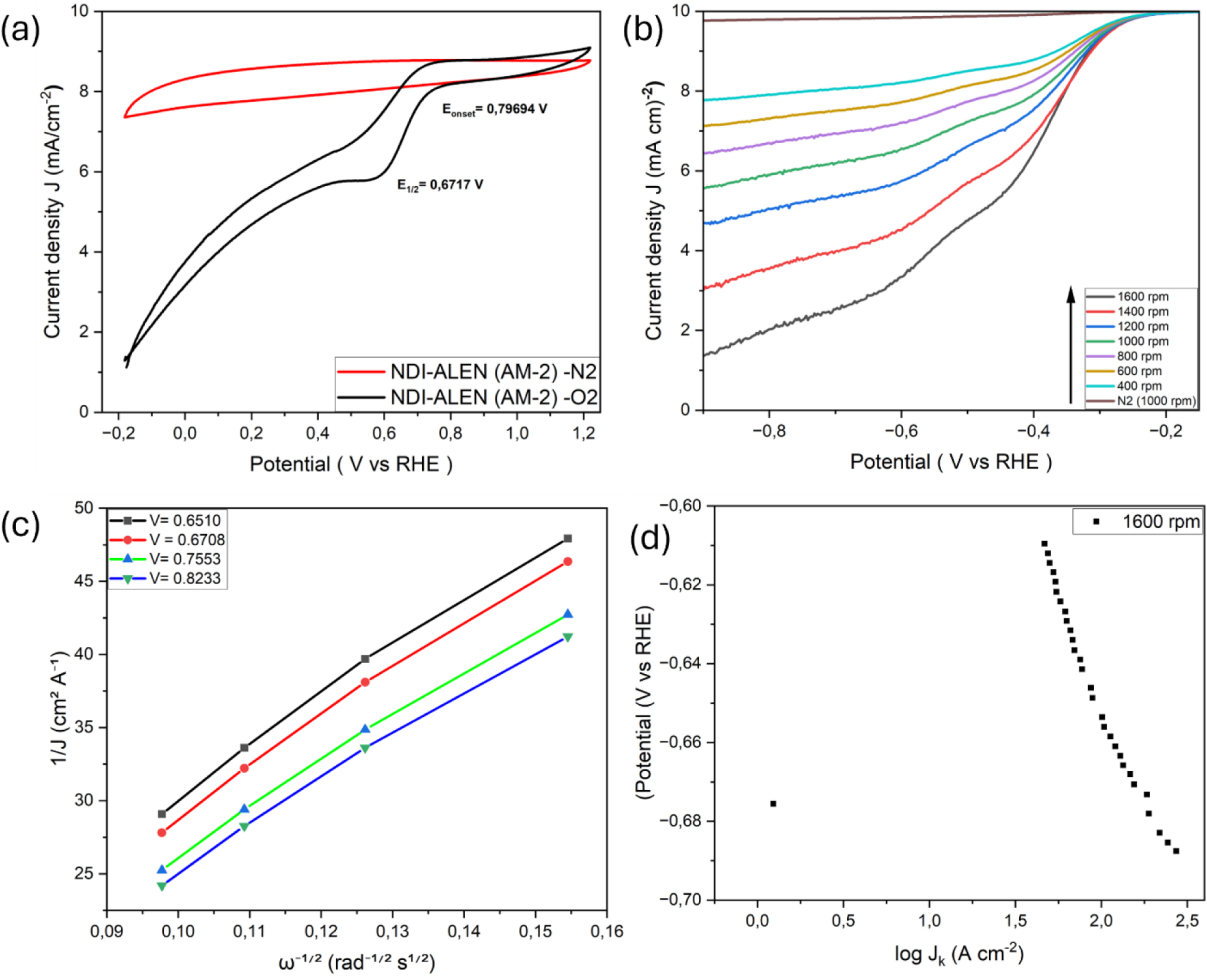
ORR characteristics of NDI-ALEN–functionalized AM-2: (a) CVs of NDI-ALEN (AM-2) in O_2_- and N_2_-saturated electrolyte, (b) RDE polarization curves at rotation rates between 400 and 1600 rpm, (c) Koutecký–Levich plots at selected potentials indicating mixed kinetic–mass-transport control, and (d) Tafel plot derived from kinetic current density.

**Figure 8:**
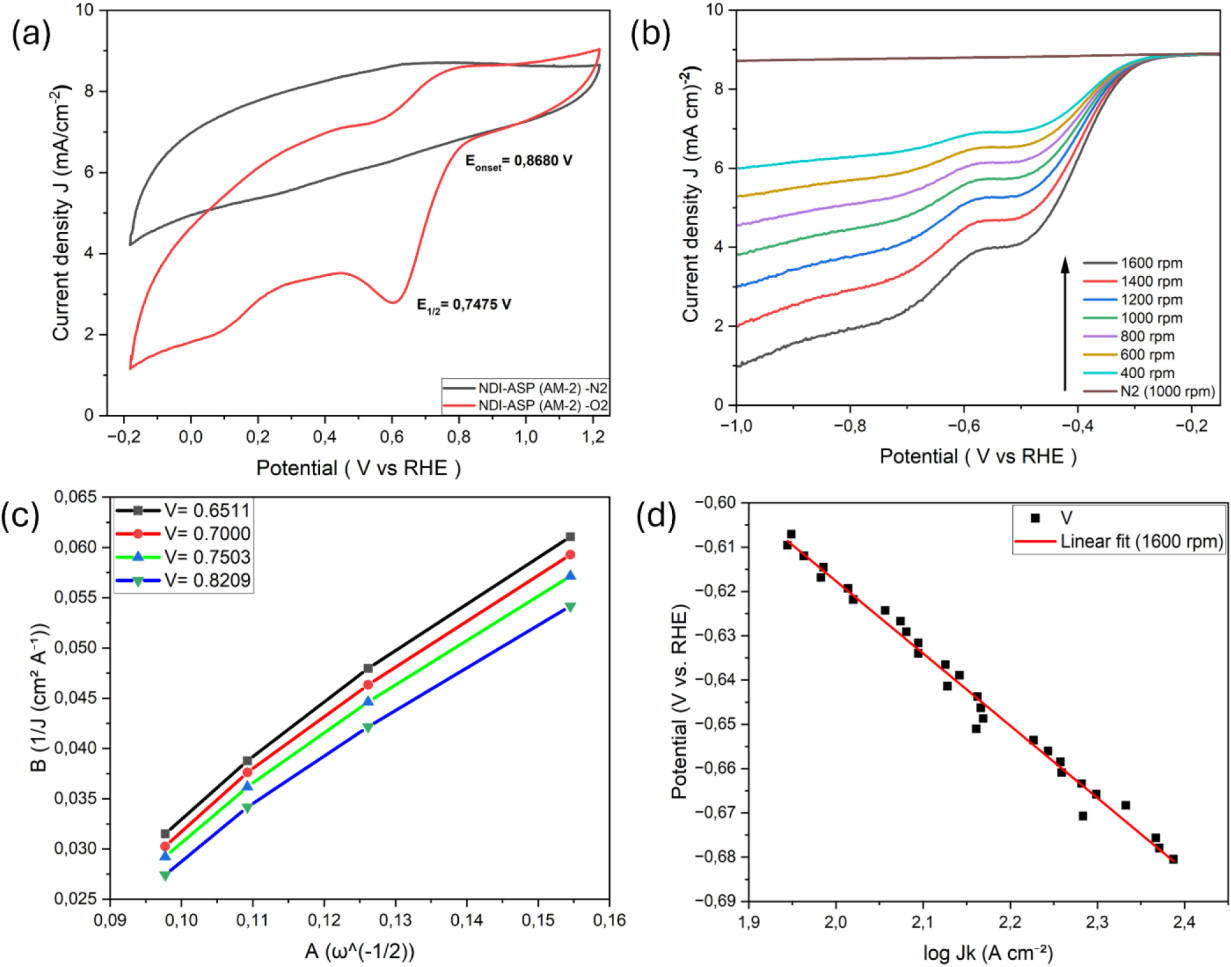
ORR performance of NDI-ASP–functionalized AM-2: (a) CVs of NDI-ASP (AM-2) recorded in O_2_- and N_2_-saturated 0.1 M KOH, (b) RDE polarization curves obtained at rotation rates from 400 to 1600 rpm, (c) Koutecký–Levich plots displaying good linearity across the analyzed potential range, and (d) Tafel plot derived from kinetic current density, indicating improved charge-transfer behavior.

Acid activation of the spent anode material led to a pronounced enhancement of the ORR performance. The sample treated with 8 M acid (AM-2) showed a positive shift in both *E*_onset_ (0.835 V) and *E*_1/2_ (0.782 V), together with a diffusion-limited current density approaching 6.0 mA cm^–2^ at 1600 rpm. Rotating disk electrode (RDE) polarization curves for AM-2 exhibited a strong and systematic increase in current density with an increasing rotation rate, confirming a significant contribution from oxygen mass transport. These improvements are attributed to oxidative activation of the graphite surface, which increases the defect density, exposes edge-plane sites, and enhances electrolyte accessibility. XPS analysis confirms significant surface chemical modification after acid activation, supporting the role of an increased defect density and oxygen-containing functionalities in enhancing ORR kinetics and mass transport.

Organic functionalization of AM-2 further modulated the ORR behavior in a manner strongly dependent on the chemical nature of the grafted molecule. BPDI-OH-Cl–functionalized AM-2 displayed a modest improvement in *E*_onset_ (0.822 V) relative to untreated AM, while *E*_1/2_ remained comparable to that of pristine graphite. Although the limiting current density was high, the rotation-rate dependence was weaker than that observed for AM-2, and Koutecký–Levich (K–L) plots exhibited significant deviation from linearity, as shown in Figure 5. This behavior is consistent with mixed kinetic control, likely arising from partial surface coverage or steric hindrance associated with the bulky π-conjugated BPDI moiety, which can restrict access to active sites and increase the charge-transfer resistance.

NDI-ALEN–functionalized AM-2 exhibited an ORR activity intermediate between BPDI-OH-Cl and AM-2, with *E*_onset_ = 0.797 V and *J*_L_ ≈ 5.7 mA cm^–2^. Figure 7 shows that the presence of phosphonate groups is expected to enhance surface polarity and electrolyte interaction; however, nonideal K–L behavior and nonlinear Tafel trends indicate mixed kinetic–diffusion control rather than an ideal diffusion-limited ORR regime. While the phosphonate groups present in NDI-ALEN can enhance surface polarity and potentially improve electrolyte interaction, phosphorus-containing carbons have generally been shown to exhibit ORR activity in alkaline media, with the specific dopant and local chemical environment governing performance trends.^35^ In our experiments, the ORR activity of NDI-ALEN (AM-2) remains lower than that of NDI-ASP (AM-2), suggesting that the nature of heteroatom functionalization—rather than the mere presence of heteroatoms—plays a key role in modulating catalytic activity. As such, quantitative electron transfer numbers were not extracted for this catalyst.

Among the synthesized catalysts, Figure 8 shows that NDI-ASP (AM-2) exhibits the most favorable overall ORR performance when the kinetic current density and charge-transfer behavior are considered, which is attributed to the effective heteroatom functionalization of the recycled graphite surface. This catalyst displayed the most positive onset potential (*E*_onset_ = 0.868 V vs RHE), comparable to that of GO, together with a high diffusion-limited current density (∼4.9 mA cm^–2^). Although the bare 8 M acid-treated AM-2 sample exhibits a more positive half-wave potential, the superior ORR activity of NDI-ASP (AM-2) is reflected in its enhanced kinetic current density, improved Koutecký–Levich linearity, and more favorable charge-transfer behavior than that of half-wave potential alone. RDE polarization curves showed strong and monotonic rotation-rate dependence, and K–L plots were linear over a wide potential range (0.65–0.82 V vs RHE), enabling physically meaningful estimation of the electron transfer number. The calculated n values increased from ∼3.4 at 0.65 V to ∼3.7 at 0.82 V, indicating a four-electron-dominated ORR behavior with a minor peroxide contribution at lower potentials.^36^ Although RRDE measurements were not performed, the n values derived from linear K–L plots indicate a predominant four-electron ORR pathway with limited peroxide accumulation (see Supporting Information for the K–L plots). Electron transfer numbers are discussed only for samples exhibiting linear Koutecký–Levich behavior; for other catalysts, deviations from ideal RDE assumptions indicate mixed kinetic–diffusion control, and quantitative *n* values are therefore not reported. In the absence of direct peroxide quantification, the ORR selectivity discussed here should be interpreted as effective rather than mechanistically exclusive.

From a mechanistic perspective, four-electron oxygen reduction is generally favored for electrochemical energy-conversion applications because it enables direct formation of H_2_O (or OH^–^ in alkaline media) while suppressing corrosive peroxide intermediates associated with two-electron pathways. For organic redox-active motifs such as naphthalene diimide derivatives, however, an apparent *n* ≈ 4 does not necessarily imply a fully concerted four-electron reduction of oxygen.^37^ Instead, ORR may proceed through an initial two-electron reduction to H_2_O_2_ (or HO_2_^–^), followed by rapid electrochemical and/or chemical consumption of peroxide species within the diffusion layer.^38^ Such sequential pathways can yield effective electron-transfer numbers close to four under rotating disk electrode conditions.^36^ While rotating ring–disk electrode measurements would be required to definitively distinguish between direct and sequential mechanisms, the combined evidence of linear Koutecký–Levich behavior, high kinetic current density, and favorable charge-transfer characteristics supports efficient oxygen reduction on NDI-ASP (AM-2) with limited peroxide accumulation near the electrode surface.

Overall, the electrochemical results reveal a clear structure–activity relationship. Untreated spent anode material exhibits kinetically limited ORR behavior, while acid activation significantly enhances the apparent activity through defect generation and improved mass transport. Organic functionalization enables further tuning of the ORR behavior: bulky aromatic units can introduce kinetic limitations, whereas nitrogen-rich functionalization (NDI-ASP) optimizes the ORR initiation and kinetics. These findings demonstrate that oxidative pretreatment primarily governs defect density and diffusion-limited activity, while targeted organic functionalization modulates surface chemistry and charge-transfer kinetics, enabling chemically tunable ORR performance from recycled graphite. In organic redox-active systems such as naphthalene diimide-based electrocatalysts, ORR activity has been shown to depend strongly on electronic structure and redox mediation rather than solely on total surface area, supporting the interpretation that heteroatom functionalization contributes to kinetic enhancement beyond porosity effects.^37^

The primary objective of the present study is to demonstrate the feasibility of upcycling spent lithium-ion battery graphite into a chemically functionalizable platform for metal-free ORR catalysis, rather than to report a fully optimized ORR catalyst. Accordingly, the electrochemical evaluation focuses on widely accepted first-order benchmarking parameters, namely the onset potential (*E*_onset_), half-wave potential (*E*_1/2_), and diffusion-limited current density (*J*_L_), obtained from CV and LSV/RDE measurements. It should be noted that while *E*_1/2_ provides insight into ORR onset behavior, comprehensive catalyst performance was evaluated based on kinetic current density, Koutecký–Levich analysis, and Tafel slopes.

Because all electrochemical measurements were conducted under identical conditions, the observed trends in the ORR parameters can be directly attributed to changes in surface chemistry induced by acid activation and molecular functionalization. Figure 9 and Table 3 highlight the synergistic effects of oxidative pretreatment and molecular functionalization on the ORR performance of the spentanode material (AM). As shown in Figure 9, functionalization increases the incorporation of nitrogen species—particularly graphitic and pyrrolic nitrogen—which enhances charge delocalization and increases the density of potential active sites. This behavior aligns with studies on N,P codoped graphene-based catalysts, which have demonstrated the critical role of heteroatom functionalities in promoting ORR activity.^39^ The comparative evaluation of ORR parameters (*E*_onset_, *E*_1/2_, and *J*_L_) further shows that the functionalized samples outperform the untreated AM. Among them, the NDI-ASP–functionalized AM-2 catalyst exhibits the most positive onset potential and a favorable diffusion-limited current density, highlighting the beneficial role of amino acid–derived nitrogen-containing surface functionalities in modulating ORR kinetics. Direct peroxide yield measurements using RRDE were not conducted in this study due to instrumentation limitations and will be included in future work to further quantify ORR selectivity. Future work will include BET surface area and electrochemically active surface area (ECSA) measurements to decouple the porosity effects from the intrinsic catalytic activity. These findings reinforce the potential of integrated chemical upcycling strategies for transforming end-of-life battery materials into efficient, metal-free ORR electrocatalysts for sustainable energy applications.

**Table 3:** Electroactivity of the Functionalized Catalyst with the Spent Anode Material (AM)

Catalysts	*E*_onset_ (V)	*E*_1/2_ (V)	*J*_L_ (mA/cm^–2^)
Anode material (AM)	0.7512	0.677	2.143
Flake graphite (FG)	0.747	0.676	3.8
Reduced graphene oxide (rGO)	0.831	0.687	2.27
Graphene oxide (GO)	0.8726	0.7544	4.762
4 M (AM-1)	0.799	0.65	2.6101
8 M (AM-2)	0.835	0.782	5.942
BPDI-OH-Cl (AM-2)	0.822	0.687	5.933
NDI-ALEN (AM-2)	0.7969	0.6717	5.665
NDI-ASP (AM-2)	0.868	0.7475	4.9306

**Figure 9:**
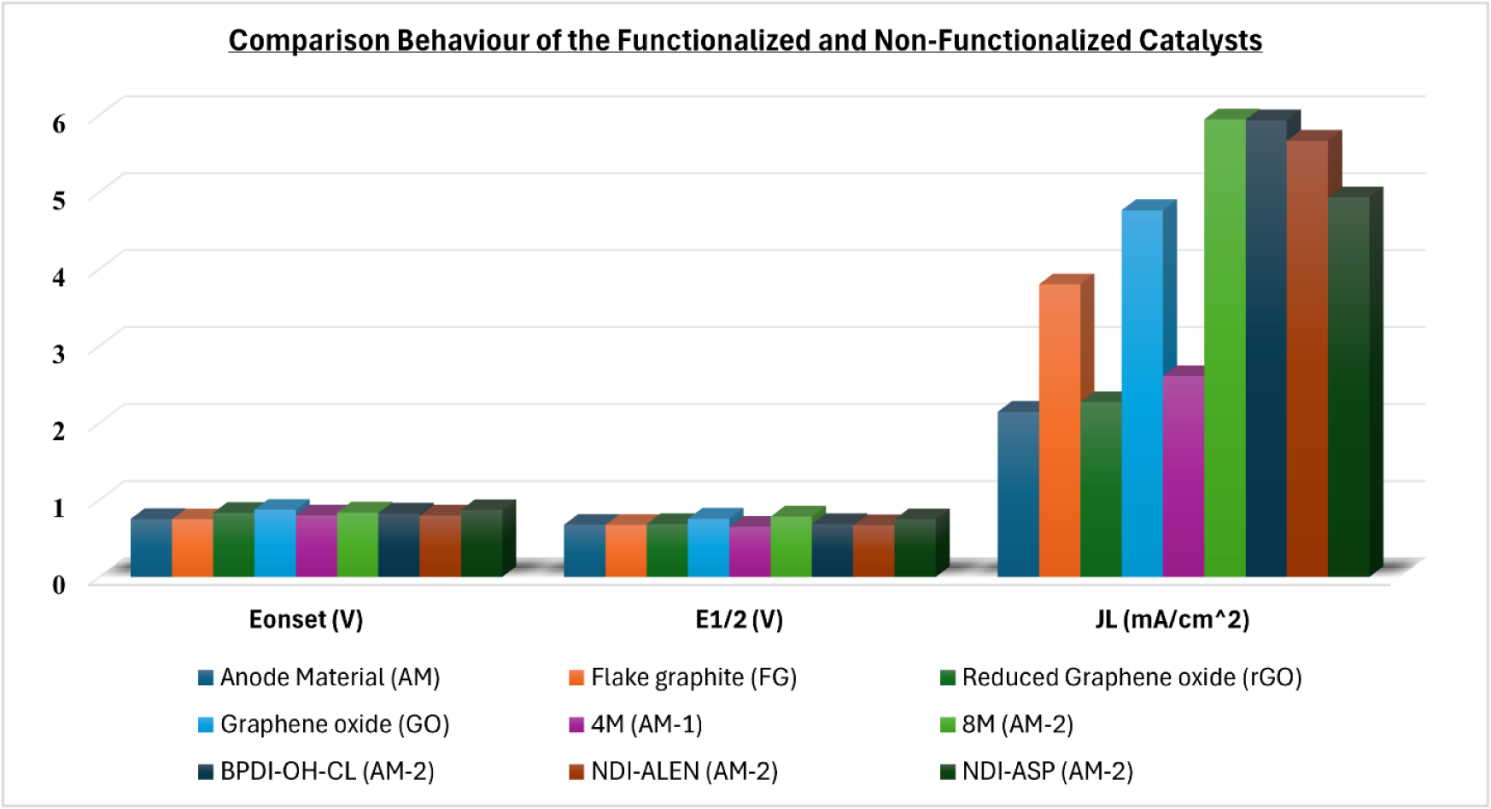
Comparison of ORR electrocatalytic parameters for various acid-treated, commercially available graphites and organic-molecule–functionalized spent anode material (AM)-based catalysts. The bar chart displays the onset potential (*E*_onset_), half-wave potential (*E*_1/2_), and diffusion-limiting current density (*J*_L_) for samples treated with H_2_SO_4_:HNO_3_ mixtures (AM-1 and AM-2) and functionalized with BPDI-OH-Cl, NDI-ALEN, and NDI-ASP. Functionalization significantly improves the ORR performance, with the NDI-ASP–modified catalyst showing the most positive onset potential and a high diffusion-limited current density, indicating enhanced ORR kinetics and charge-transfer behavior arising from nitrogen-containing surface functionalities. The results underscore the importance of combining oxidative treatment with strategic molecular functionalization to develop efficient, metal-free ORR catalysts from recycled battery materials.

## 4 Conclusion

In this study, spent anode material (AM) from lithium-ion batteries was upcycled into metal-free oxygen reduction reaction (ORR) electrocatalysts through a two-step approach involving acid activation and organic surface functionalization. Oxidative treatment with an 8 M H_2_SO_4_/HNO_3_ mixture increased the defect density and exposed a greater number of reactive sites compared to milder 4 M conditions, leading to substantial enhancement of apparent ORR activity. CV, LSV, and RDE measurements confirmed positive shifts in onset and half-wave potentials for the acid-activated materials.

Among the functionalized catalysts, NDI-ASP (AM-2) exhibits the most favorable ORR kinetics and charge-transfer behavior, despite the bare AM-2 sample displaying the highest half-wave potential. This distinction highlights that defect generation and molecular functionalization influence different ORR descriptors: acid activation primarily governs apparent activity, while nitrogen-containing surface functionalities introduced by NDI-ASP promote improved reaction kinetics and pathway selectivity. Functionalization with NDI-alendronic acid (phosphonate-containing) and BPDI-OH-Cl (π-conjugated) resulted in more moderate performance changes, underscoring the importance of both heteroatom chemistry and electronic structure in the rational design of carbon-based ORR catalysts.

Overall, this work demonstrates a scalable and circular-economy–oriented route for valorizing spent graphite from end-of-life lithium-ion batteries into chemically tunable, metal-free ORR catalysts. The observed performance trends are consistent with reports of heteroatom-doped graphene systems approaching the activity of established carbon-based catalysts. While the present methodology involves chemically intensive steps, it establishes a robust proof-of-concept foundation for future optimization toward lower-impact activation strategies, greener solvent systems, and deeper kinetic and durability analyses.

## Supplementary Material



## Notes

Copies of the NMR spectra of the NDI analogues; ^1^H and ^13^C spectra (PDF)

## Abbreviations

ORR: Oxygen Reduction Reaction
PEMFC: Proton Exchange Membrane Fuel Cell
LIB: Lithium-Ion Battery
NMC: Nickel Manganese Cobalt (commonly used in battery cathodes)
AM: Anode Material (specifically, spent graphite anode from LIBs)
GO: Graphene Oxide
rGO: Reduced Graphene Oxide
FG: Flake Graphite
*E*_onset_: Onset Potential
*E*_1/2_: Half-Wave Potential
*J*_L_: Diffusion-Limiting Current Density
CV: Cyclic Voltammetry
LSV: Linear Sweep Voltammetry
RDE: Rotating Disk Electrode
XPS: X-ray Photoelectron Spectroscopy
SEM: Scanning Electron Microscopy
EDX: Energy Dispersive X-ray Spectroscopy
XRD: X-ray Diffraction
XRF: X-ray Fluorescence
BPDI: 2,2′-Bis­(2-(dimethylamino)­ethyl)-[5,5′-biisoindoline]-1,1′,3,3′-tetraone
BPDI-OH-Br: Hydroxylated BPDI with Bromide Counterion
BPDI-OH-Cl: Hydroxylated BPDI with Chloride Counterion
NDI-ASP: Aspartic Acid–Functionalized Naphthalene Diimide
NDI-ALEN: Alendronic Acid–Functionalized Naphthalene Diimide
HRMS: High-Resolution Mass Spectrometry
NMR: Nuclear Magnetic Resonance
TMSP-*d*_4_: 3-(Trimethylsilyl)­propionic-2,2,3,3-*d*_4_ Acid (used as an NMR reference)
