# Supplementary material for: Enhanced ORR Activity of Modified Recycled Graphite-Based Anode Materials
Source: ACS Omega. 2026 Mar 11;11(11):18155–67. doi: 10.1021/acsomega.5c13315 (PMC13019378; doi:10.1021/acsomega.5c13315)

Supporting Information

Enhanced ORR Activity of Modified Recycled Graphite-Based Anode Material

Sukanya Sukanya[a], Kimia Hoseinzade[a], Frederik Bettels[b], Lin Zhang[b], René Wilhelm[a]*

[a]*Institute of Organic Chemistry, Clausthal University of Technology, Leibnizstrasse 6, 38678 Clausthal-Zellerfeld, Germany*

[b]*Institute of Solid State Physics and Laboratory of Nano and Quantum Engineering, Leibniz University Hannover, Appelstrasse 2, 30167 Hannover, Germany*

*E-mail: rene.wilhelm@tu-clausthal.de

Contents

Figure S1: E CV and LSV results for GO, rGO, FG, AM (O2 vs N2): 2

Table S1: Bulk elemental composition of samples AM, AS-1, AA-1, and P-1 determined by EDX analysis...4

^{1}H NMR spectrum of compound (BPDI): 6

^{13}C NMR spectrum of compound (BPDI): 7

^{1}H NMR spectrum of compound (BPDI-OH-Br): 8

^{13}C NMR spectrum of compound (BPDI-OH-Br): 9

^{1}H NMR spectrum of compound (BPDI-OH- Cl): 10

^{13}C NMR spectrum of compound (BPDI-OH- Cl): 11

^{1}H NMR spectrum of compound (NDI-ASP): 12

^{13}C NMR spectrum of compound (NDI-ASP): 13

^{1}H NMR spectrum of compound (NDI-ALEN): 14

^{13}C NMR spectrum of compound (NDI-ALEN): 15

This Supporting Information provides additional electrochemical data, elemental analysis, and spectroscopic characterization supporting the conclusions of the main manuscript.

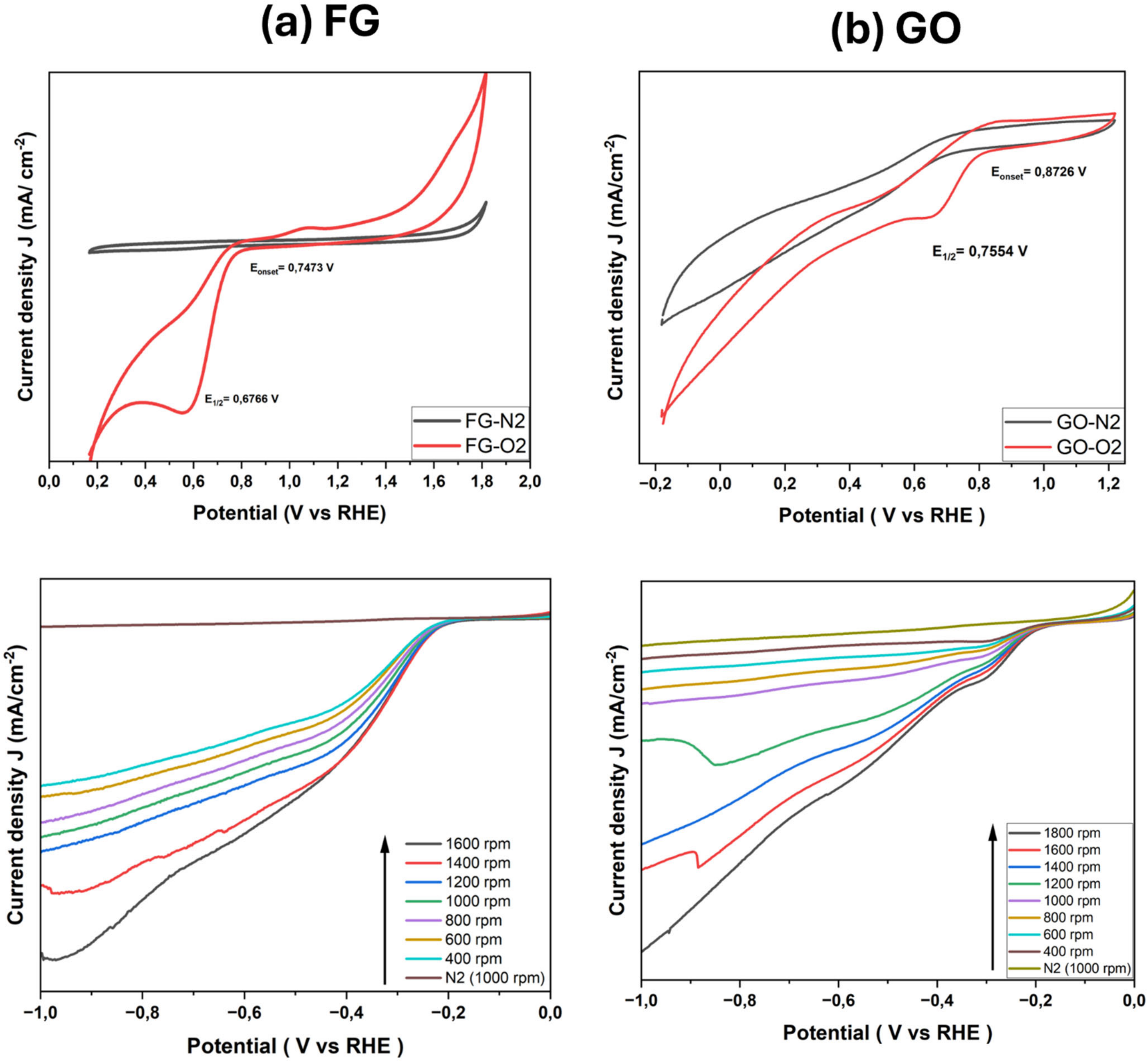

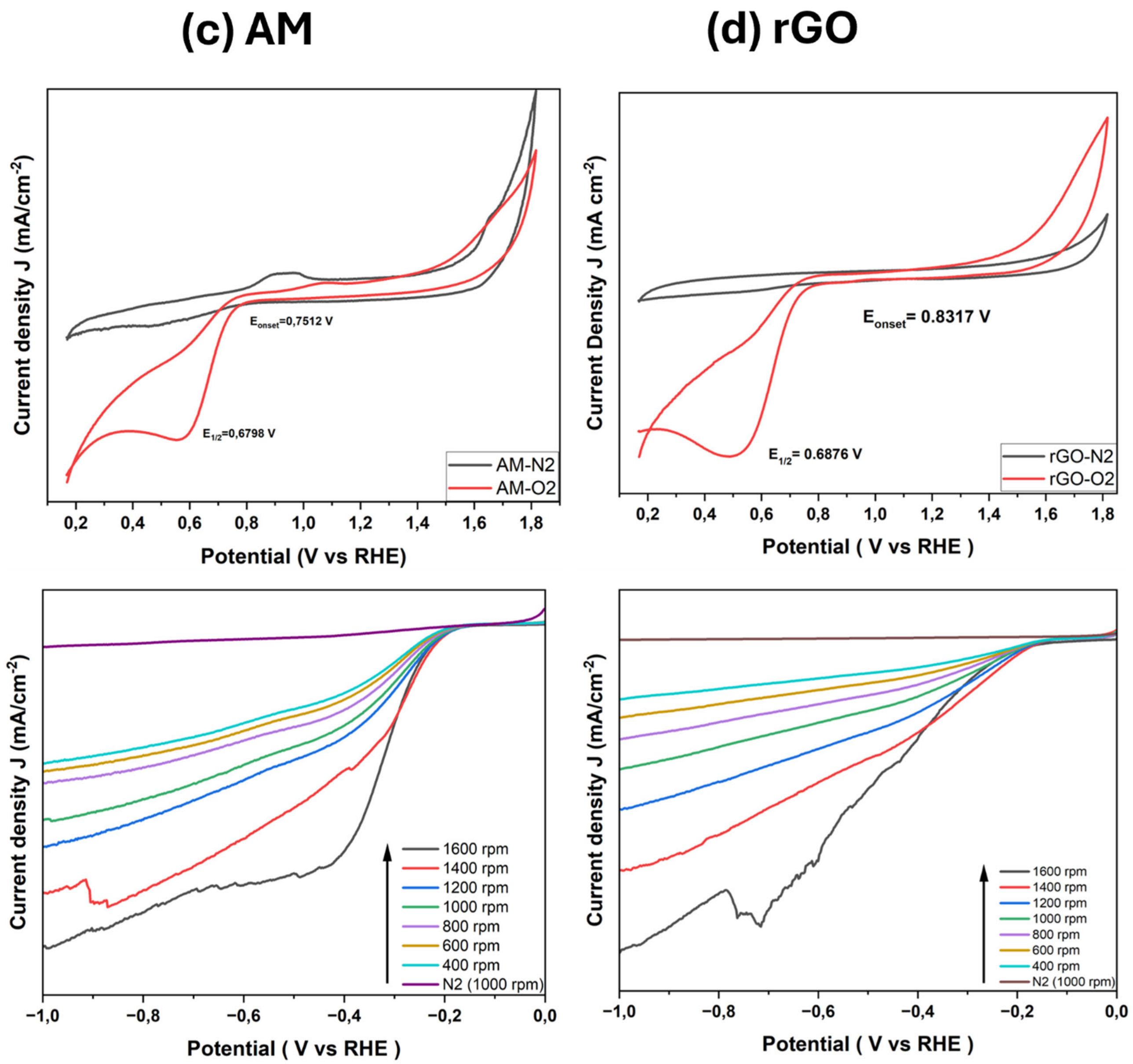

Figure S1: Electrochemical evaluation of flake graphite (FG), graphene oxide (GO), anode material (AM), and reduced graphene oxide (rGO) in 0.1 M KOH. The top panels show CV responses recorded under N_2- and O_2-saturated conditions, where onset potential (E_{onset}) and half-wave potential ($E_{1/2}$) provide a measure of ORR activity: (a) FG, (b) GO, (c) AM, and (d) rGO. The lower panels display LSV curves at rotation rates between 400 and 1800 rpm, illustrating the rise in current density with faster rotation, in line with improved mass transport during oxygen reduction.

Table S1. Bulk elemental composition of samples AM, NDI-ASP (AM-2), NDI-ALEN (AM-2), and BPDI-OH (AM-2) determined by EDX analysis

Sample	C (at%)	O (at%)	Cu (at%)
**AM**	96.07	3.33	0.6
**AS-1**	98.28	1.72	—
**AA-1**	98.01	1.99	—
**P-1**	99.3	0.7	—

Note: "—" indicates values below the detection limit of EDX. EDX is a bulk-sensitive technique with limited sensitivity for light heteroatoms (N, O, P) at low surface concentrations; therefore, surface chemical functionalization was evaluated primarily using XPS.

Calculation of NDI-ASP (AM-2) number of electron transfer (*n*):

Constants used: For ORR in O_2-saturated 0.1 M KOH at 25 °C:

- $F = 96485\ \mathrm{C\ mol^{-1}}$
- $C_{O_2} = 1.2 \times 10^{-6}\ \mathrm{mol\ cm^{-3}}$
- $D_{O_2} = 1.9 \times 10^{-5}\ \mathrm{cm^2 s^{-1}}$
- $\nu = 0.01\ \mathrm{cm^2 s^{-1}}$

(1) K–L constant:

$$B = 0.62\, nFC_{O_2}D_{O_2}^{2/3}\nu^{-1/6}$$

(2) Numerically:

$$B = 0.121\, n(\mathrm{A\ cm^{-2}\ rad^{-1/2} s^{1/2}})$$

(3) Thus:

$$n = \frac{1}{0.121 \times (\text{slope of } 1/J \text{ vs } \omega^{-1/2})}$$

Linear fits and calculated *n*

V = 0.6511 V

Slope = 0.301 $cm^2\ A^{-1}\ (rad^{-1/2}\ s^{1/2})^{-1}$

$$n = \frac{1}{0.121 \times 0.301} = \mathbf{3.44}$$

V = 0.7000 V

Slope = 0.291$cm^2\ A^{-1}\ (rad^{-1/2}\ s^{1/2})^{-1}$

$$n = \frac{1}{0.121 \times 0.291} = \mathbf{3.52}$$

V = 0.7503 V

Slope = 0.281 $cm^2\ A^{-1}\ (rad^{-1/2}\ s^{1/2})^{-1}$

$$n = \frac{1}{0.121 \times 0.281} = \mathbf{3.58}$$

V = 0.8209 V

Slope = 0.266 $cm^2\ A^{-1}\ (rad^{-1/2}\ s^{1/2})^{-1}$

$$n = \frac{1}{0.121 \times 0.266} = \mathbf{3.70}$$

Table S2. Linear fit -calculated n for NDI-ASP (AM-2)

**Potential (V vs RHE)**	**Electron transfer number (*n*)**
0.6511	3.44
0.7000	3.52
0.7503	3.58
0.8209	3.70

Figure S2: ^{1}H NMR spectrum of compound (BPDI):

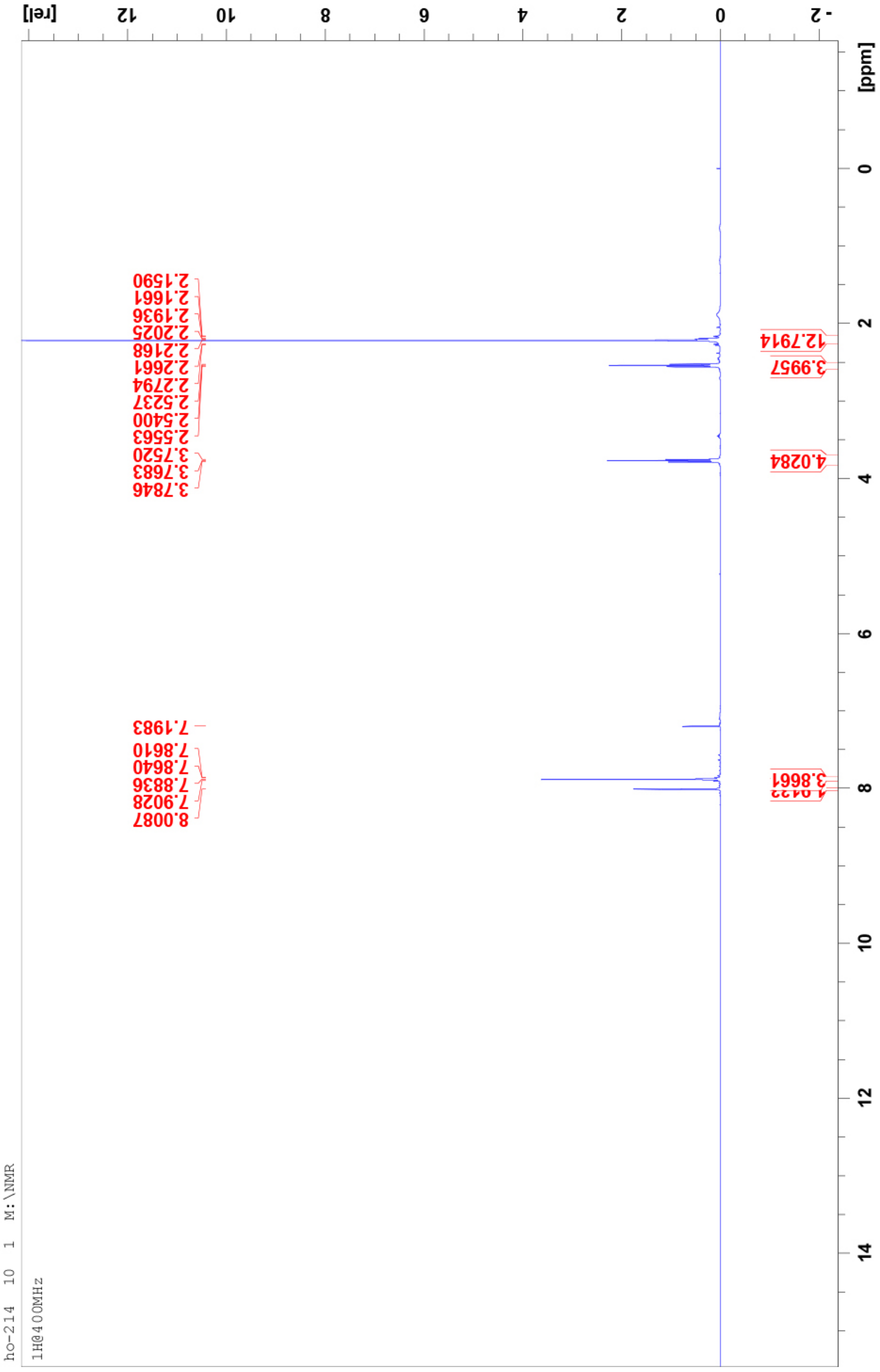

Figure S3: ^{13}C NMR spectrum of compound (BPDI):

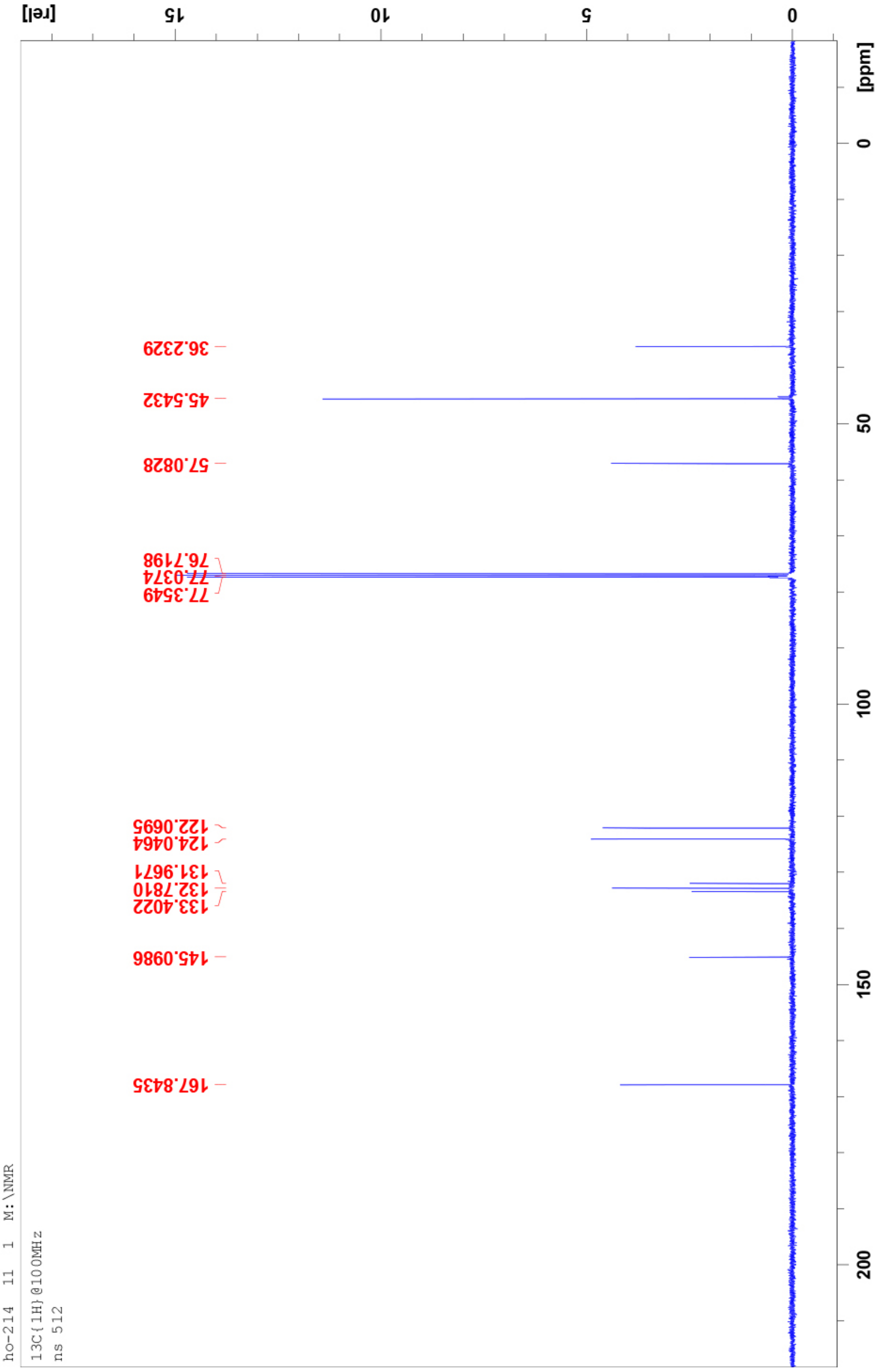

Figure S4: ^{1}H NMR spectrum of compound (BPDI-OH-Br):

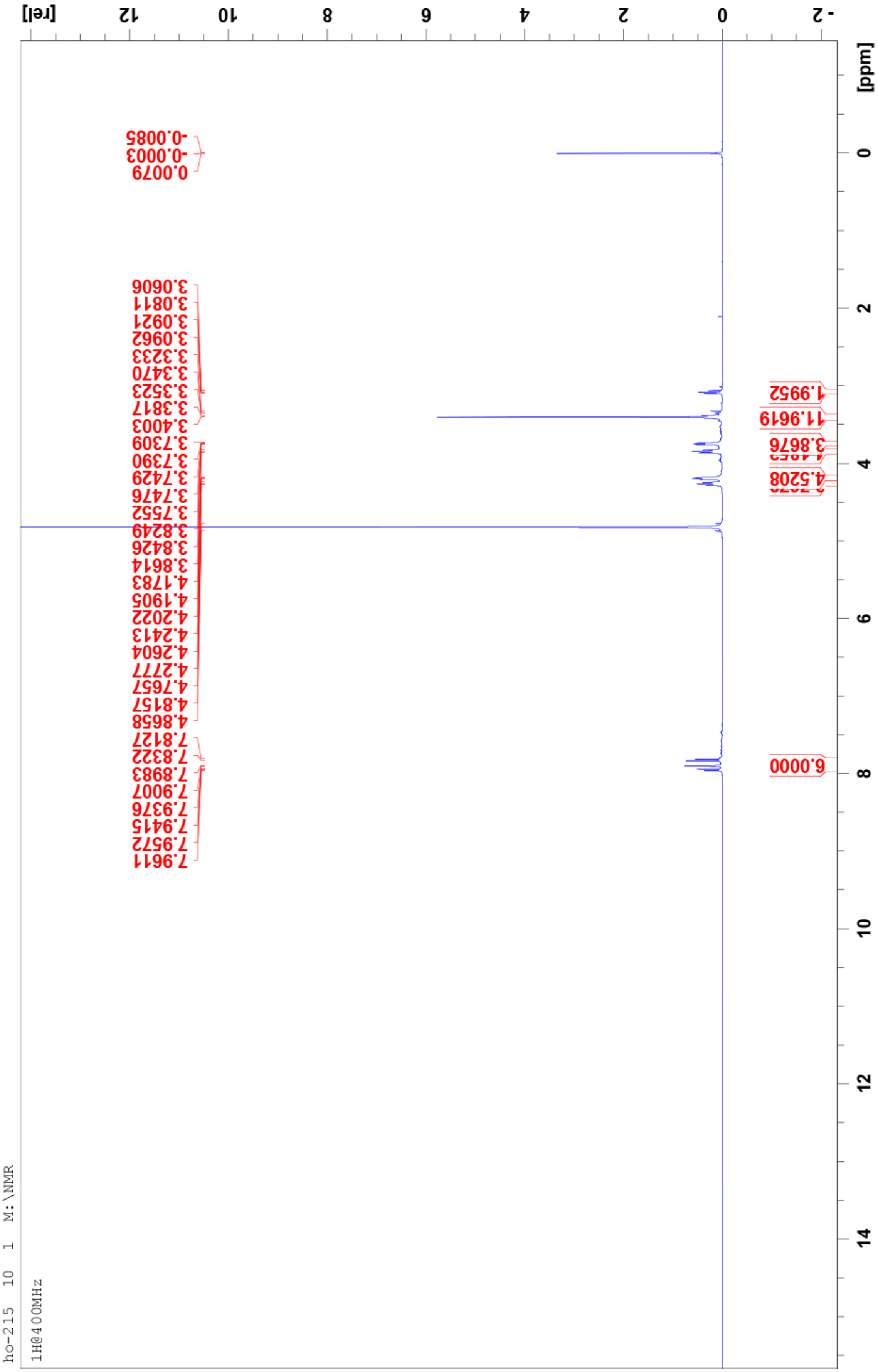

Figure S5: ^{13}C NMR spectrum of compound (BPDI-OH-Br):

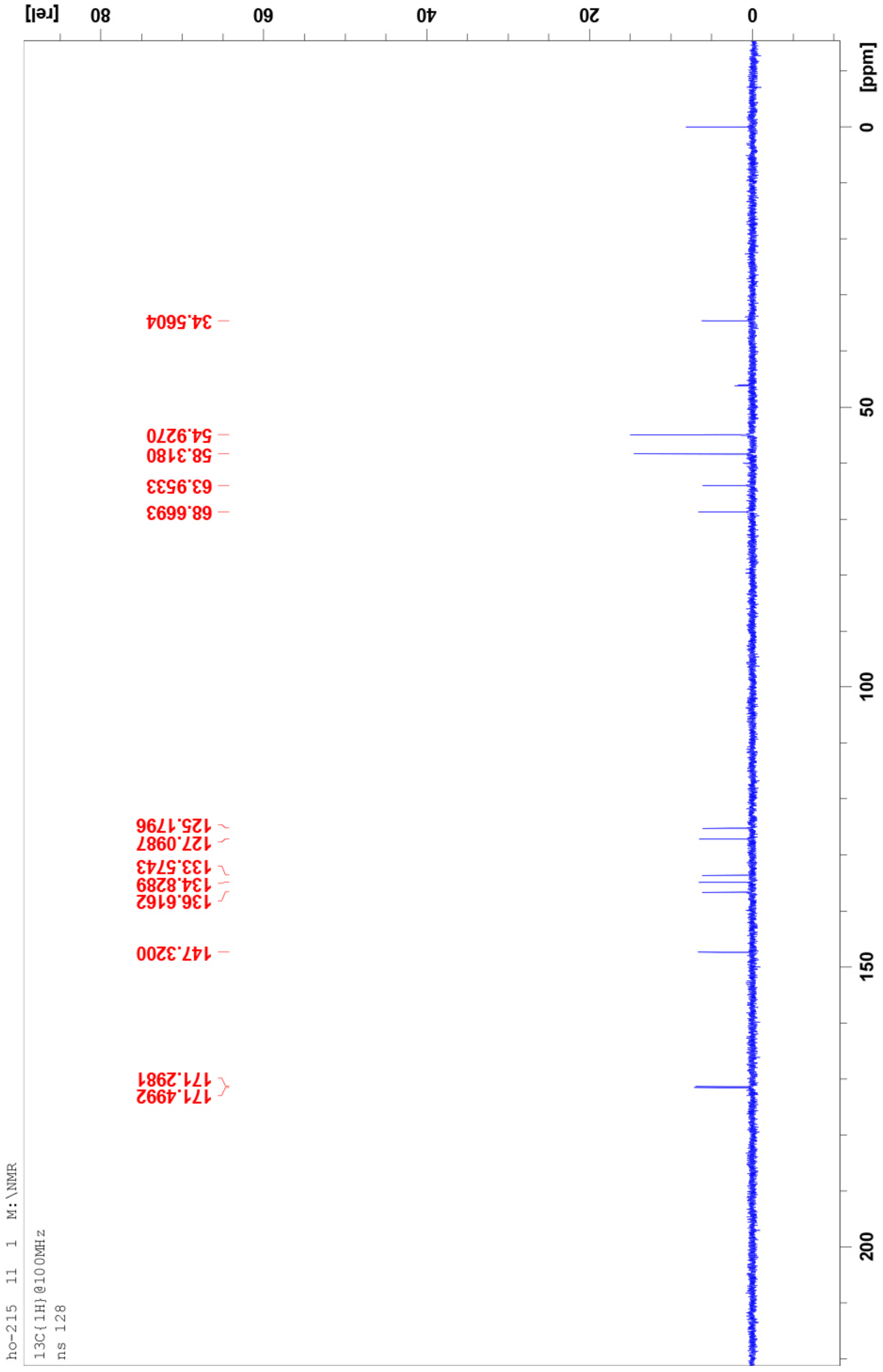

Figure S6: ^{1}H NMR spectrum of compound (BPDI-OH- Cl):

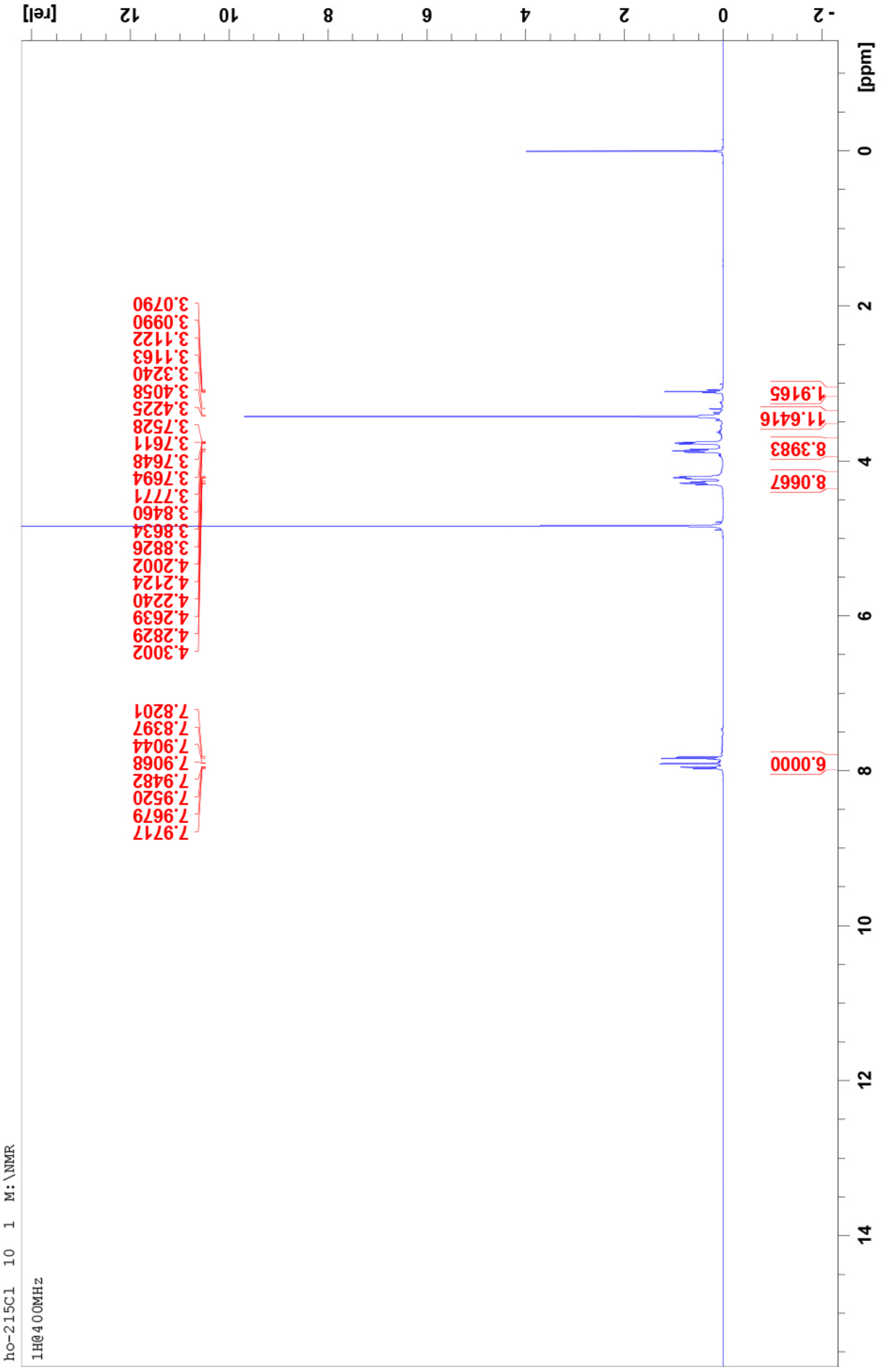

Figure S7: ^{13}C NMR spectrum of compound (BPDI-OH- Cl):

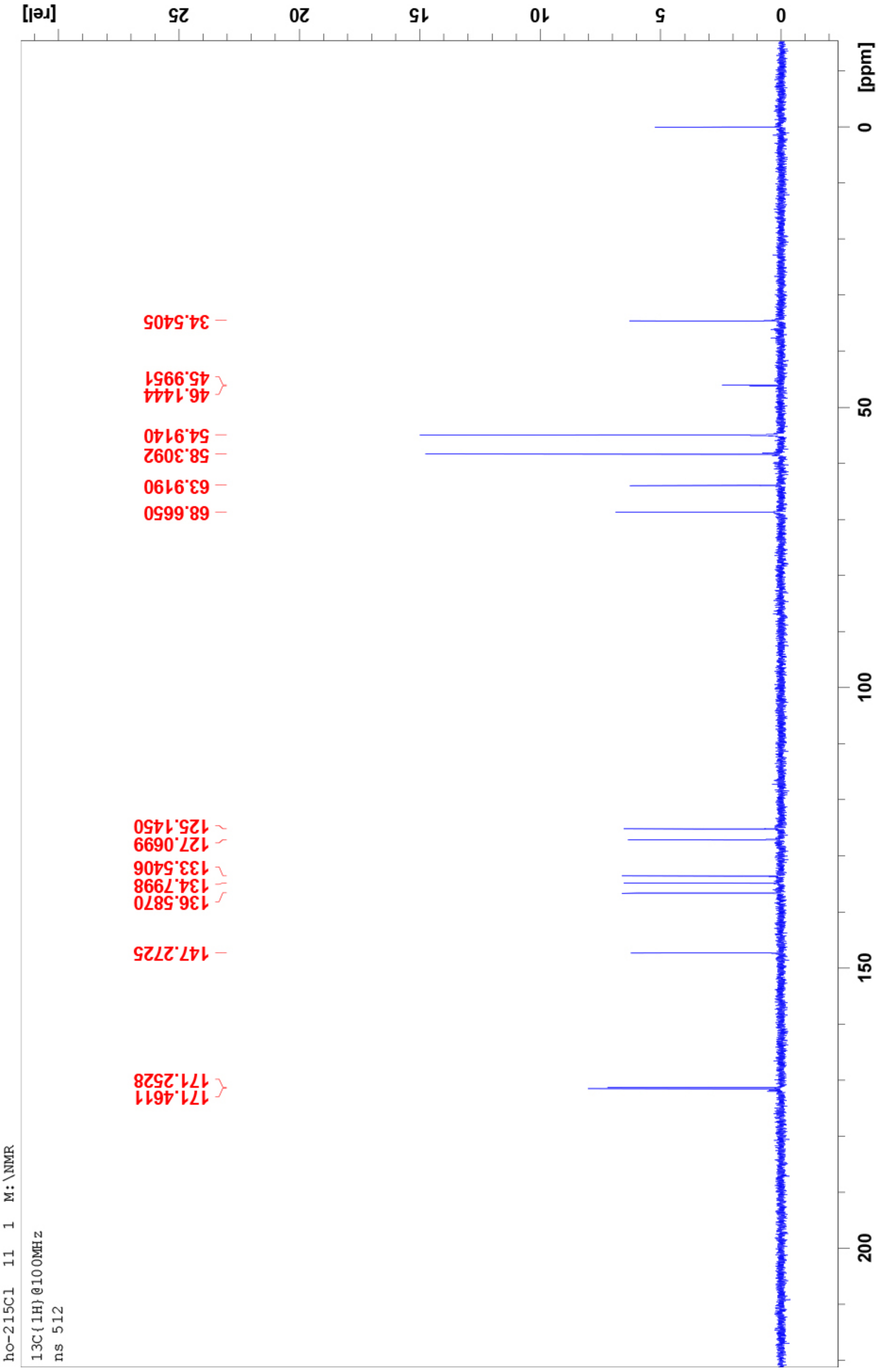

Figure S8: ^{1}H NMR spectrum of compound (NDI-ASP):

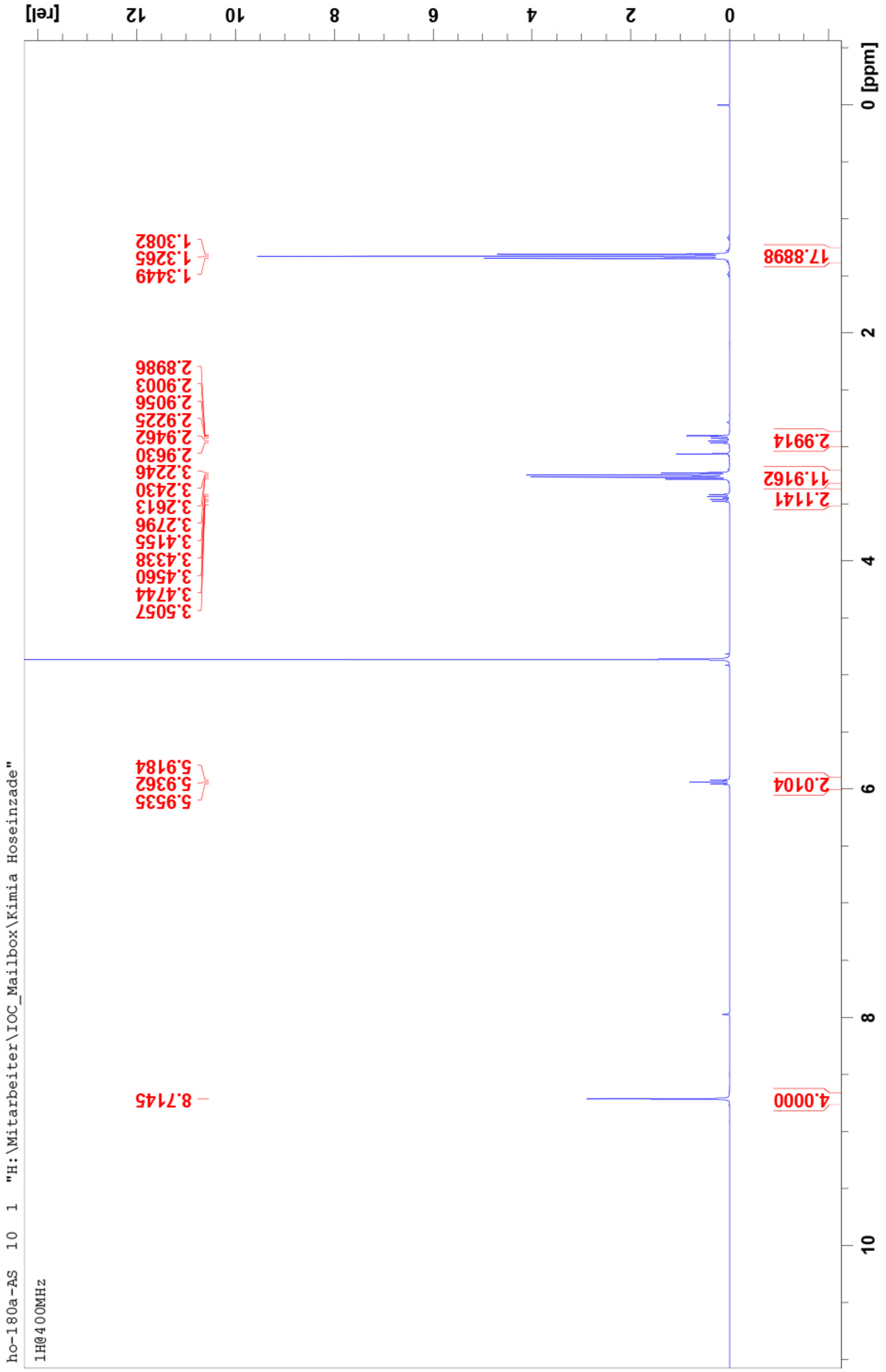

Figure S9: ^{13}C NMR spectrum of compound (NDI-ASP):

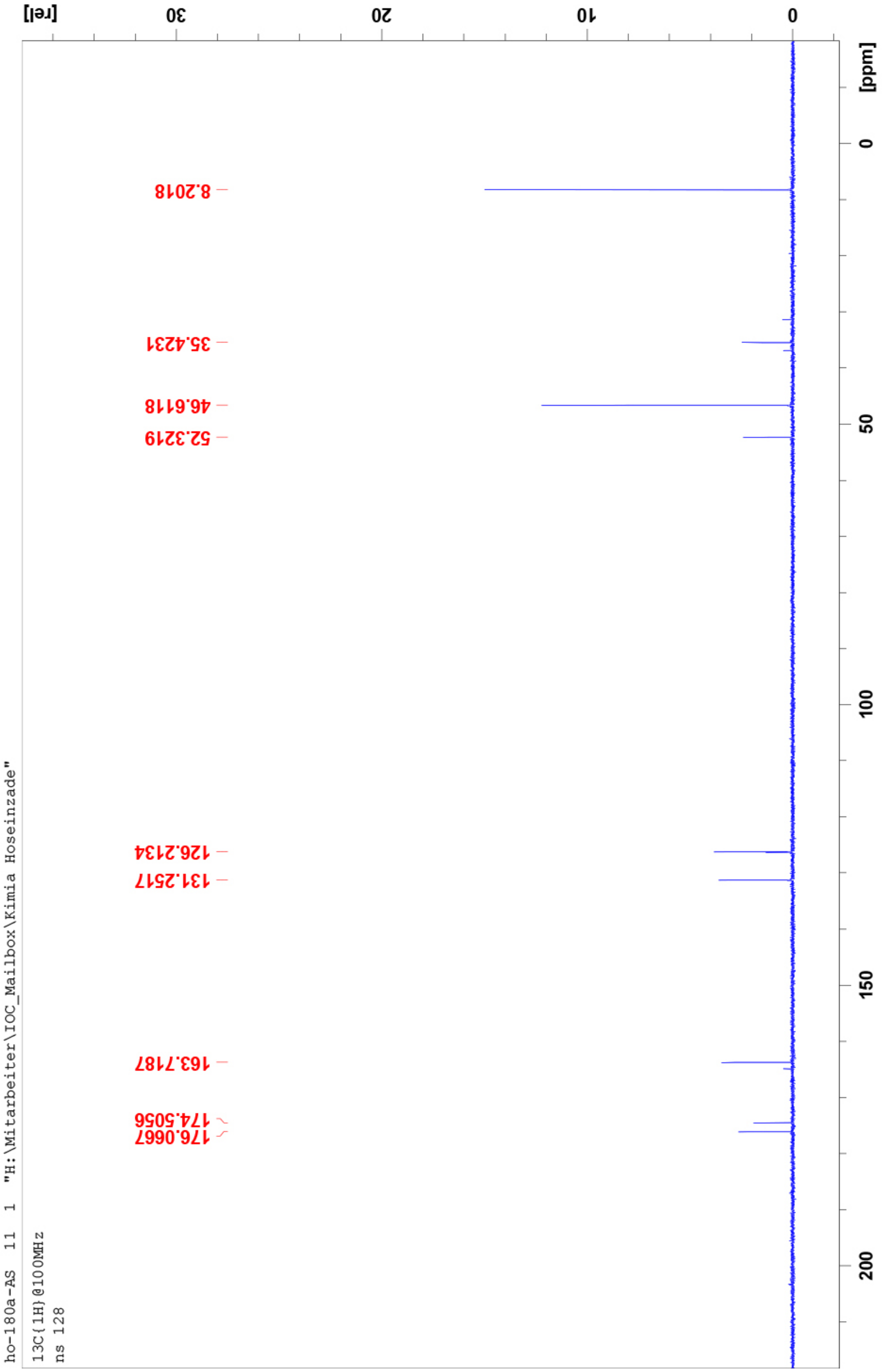

Figure S10: ^{1}H NMR spectrum of compound (NDI-ALEN):

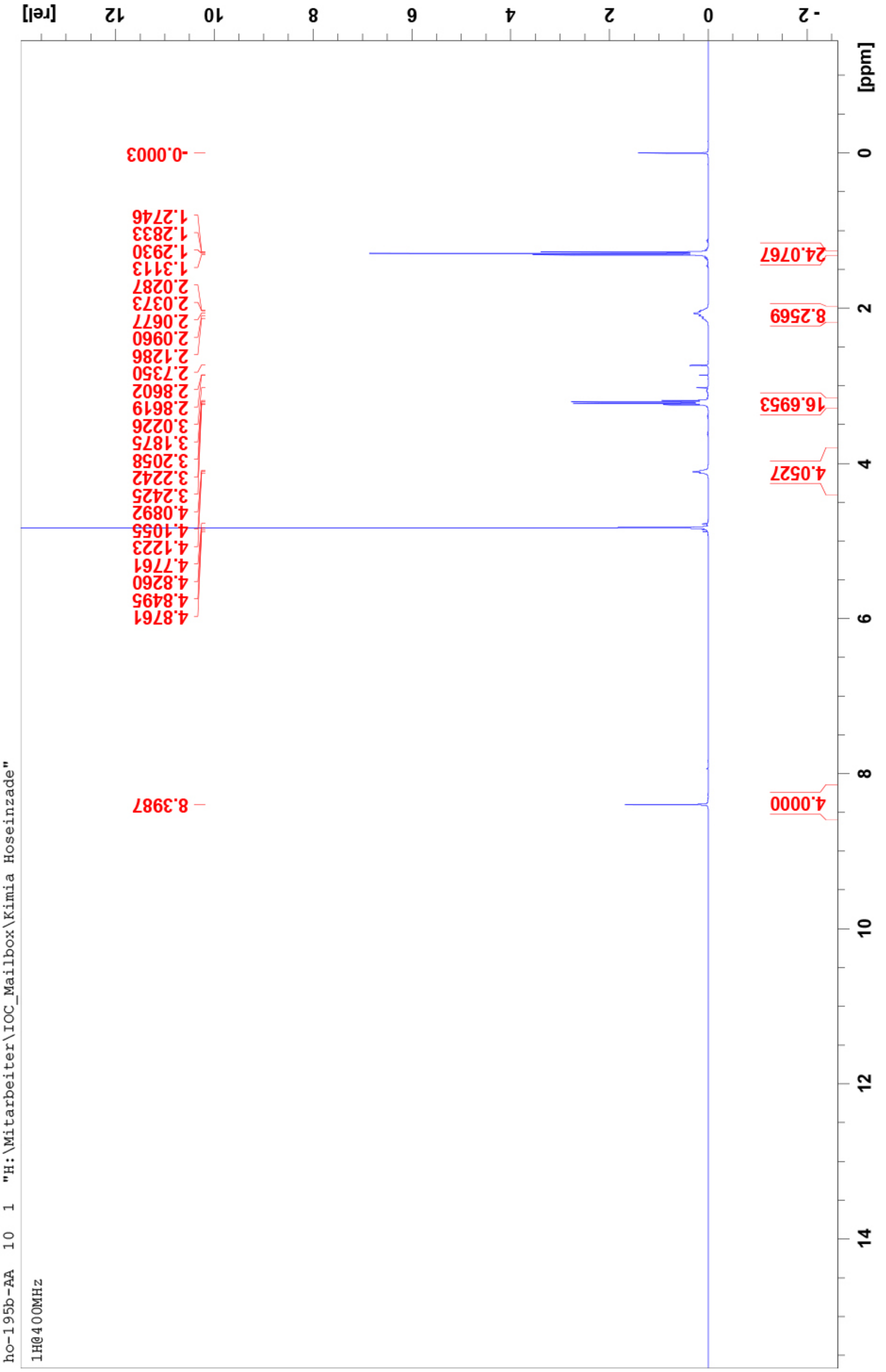

Figure S11: ^{13}C NMR spectrum of compound (NDI-ALEN):

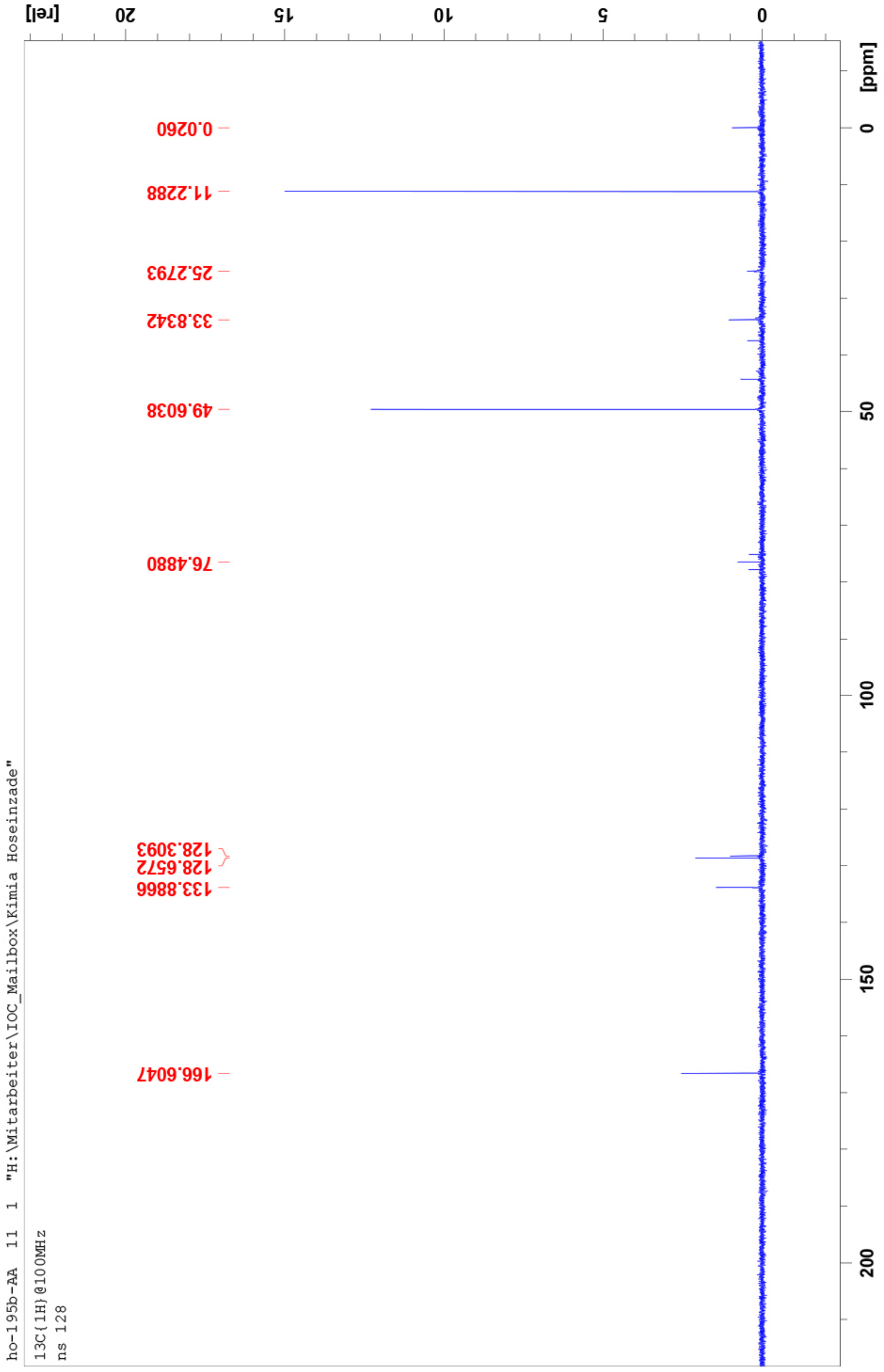

Supplement: Supplementary file 1 [file ao5c13315_si_001.pdf]
